# Insight into the molecular recognition mechanism of the coactivator NCoA1 by STAT6

**DOI:** 10.1038/s41598-017-17088-5

**Published:** 2017-12-04

**Authors:** Luigi Russo, Karin Giller, Edith Pfitzner, Christian Griesinger, Stefan Becker

**Affiliations:** 1 Department for NMR based Structural Biology, Max Planck Institute for Biophysical Chemistry, Am Fassberg 11, 37077 Göttingen, Germany; 2 Friedrich-Schiller-University Jena, Institute of Biochemistry and Biophysics, Philosophenweg 12, 07743 Jena, Germany; 3 Present Address: Department of Environmental, Biological and Pharmaceutical Sciences and Technologies, University of Campania “Luigi Vanvitelli”, 81100 Caserta, Italy; 4 Present Address: University of Kassel, Mönchebergstr. 19, 34109 Kassel, Germany

## Abstract

Crucial for immune and anti-inflammatory cellular responses, signal transducer and activator of transcription 6 (STAT6) regulates transcriptional activation in response to interleukin-4 and -13 -induced tyrosine phosphorylation by direct interaction with coactivators. The interaction of STAT6 with nuclear coactivator 1 (NCoA1) is mediated by a short region of the STAT6 transactivation domain that includes the motif LXXLL and interacts with the PAS-B domain of NCoA1. Despite the availability of an X-ray structure of the PAS-B domain/ Leu^794^-Gly^814^-STAT6 complex, the mechanistic details of this interaction are still poorly understood. Here, we determine the structure of the NCoA1^257–385^/STAT6^783–814^ complex using Nuclear Magnetic Resonance (NMR) and X-ray crystallography. The STAT6^783–814^ peptide binds with additional N-terminal amino acids to NCoA1^257–385^, compared to the STAT6^794–814^ peptide, explaining its higher affinity. Secondary and tertiary structures existing in the free peptide are more highly populated in the complex, suggesting binding by conformational selection.

## Introduction

STAT6 belongs to a family of transcription factors known as the signal transducers and activators of transcription (STAT). STAT family members share a similar protein structure, which is essential for their activation and function. They are composed of an N-terminal coiled-coil domain^1^, a centrally located DNA-binding domain^2^, a linker region, an SH2 domain for dimerization^3^ and a transactivation domain at the C-terminus^4^. STAT proteins mediate signaling from activated cytokine receptors to the nucleus^5^. After phosphorylation at a specific tyrosine by a receptor associated Janus kinase, STATs form homo- or heterodimers and translocate into the nucleus where they modulate transcription after binding to specific DNA sequence elements^4,5^. STAT6 becomes activated in response to IL-4 and IL-13 and mediates most of the gene expression regulated by these cytokines^6^.

**Figure 1:**
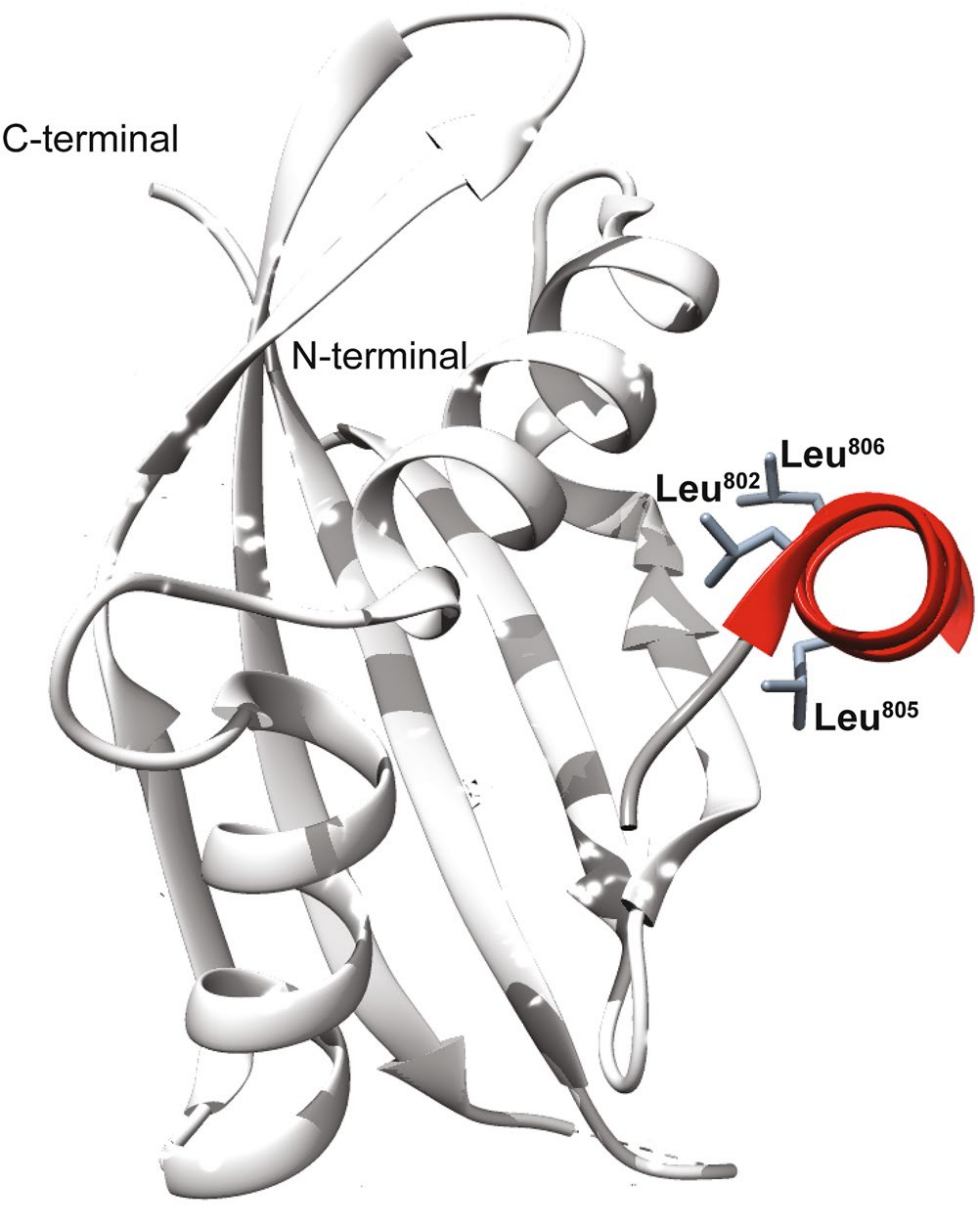
Overview of the NCoA1/STAT6^794–814^ X-ray structure (PDB ID: 1OJ5). Ribbon drawing representation of the NCoA1/STAT6^794–814^ complex. The helical region containing the LXXLL motif of the STAT6 derived peptide is depicted in red. The side chains of the STAT6 residues Leu^802^, Leu^805^ and Leu^806^ are shown as sticks.

**Figure 2:**
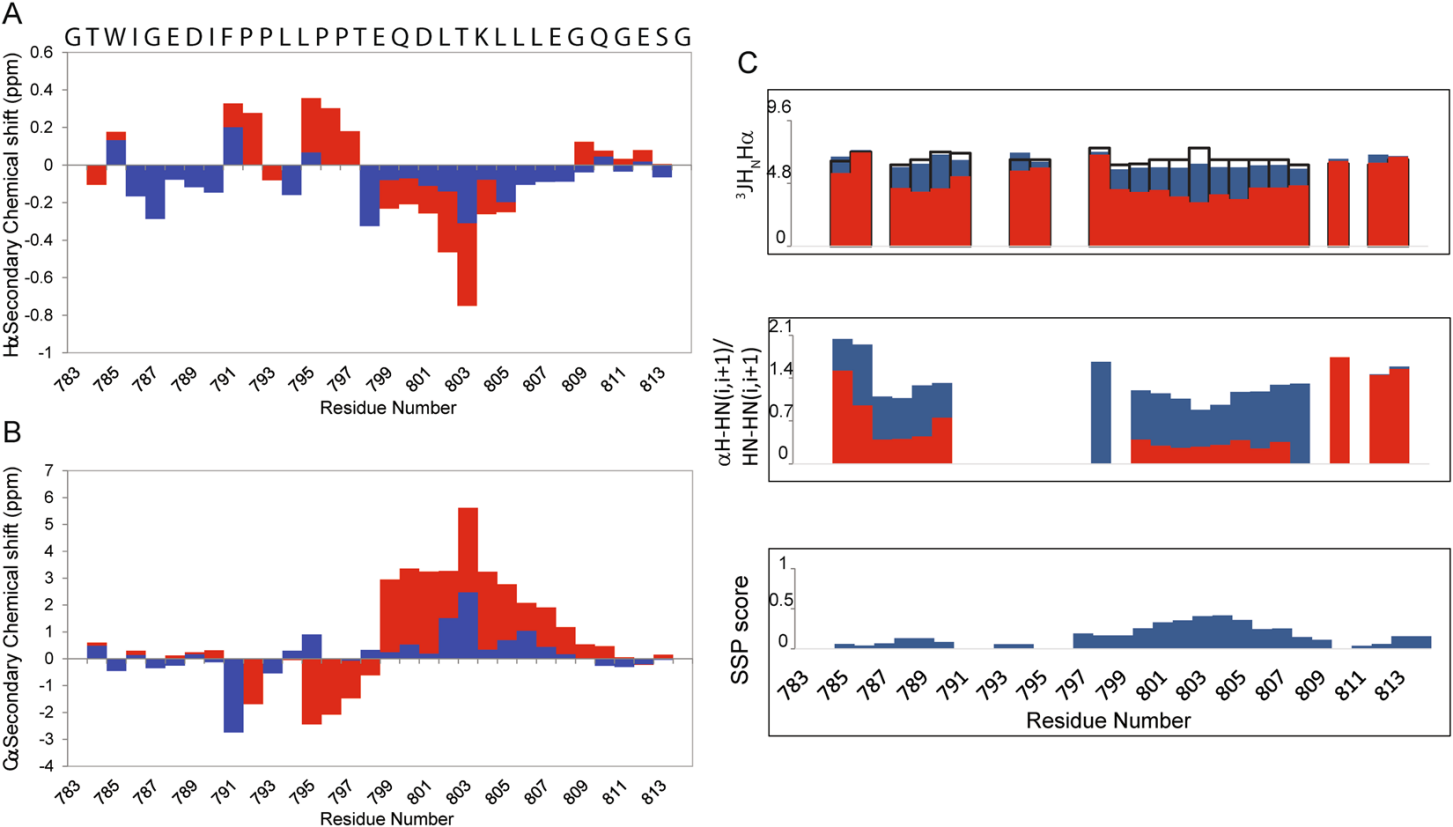
Secondary structure propensity analysis of STAT6^783–814^. (**A,B**) Secondary chemical shifts of Hα (**A**) and Cα (**B**) for the STAT6^783–814^ peptide in the free (blue) and bound (red) state. (**C**) ^3^JH_N_Hα coupling constants (*upper*) and the intensity ratio of αH-HN(i,i+1)/HN-HN(i,i+1) NOEs (*middle*) for the STAT6^783–814^ peptide in the free (blue) and bound (red) state. The open bars indicate the reference ^3^JH_N_Hα values in the random coil conformation^17^ and the reference values of the intensity ratio of αH-HN(i,i+1)/HN-HN(i,i+1) NOEs are: 1.4 for random coil, 0.25 for α-helix and 55 for β-strand^17^. The SSP score (Secondary structural propensity) (*lower*) of the free STAT6^783–814^ peptide is also reported.

By direct interaction with specific parts of its transactivation domain, STAT6 recruits the co-activators p300/CBP and NCoA1 (also called steroid receptor coactivator-1, SRC-1), which are essential for transcriptional activation^7^. In particular, the interaction between STAT6 and NCoA1 is modulated by a short region of the transactivation domain that includes the motif LXXLL (where L is leucine and X is any amino acid)^7^. The crystal structure of a STAT6-derived peptide (Leu^794^-Gly^814^) in complex with the NCoA1 PAS-B domain^257–385^ (PDB ID: 1OJ5) (Fig. 1) revealed that the leucine side-chains of the motif (Leu^802^, Leu^805^ and Leu^806^), are deeply embedded into a hydrophobic groove on the surface of NCoA1^8^. More recently, Robinson and coworkers^9^ demonstrated by fluorescence polarization (FP) binding assays that a peptide comprising STAT6 residues 783–814 (for sequence see Fig. 2) binds about 6.5 to 8 times stronger than Leu^794^- Gly^814^ (K_d_ = 0.04 µM vs. 0.32 µM from direct FP, K_i_ = 0.04 µM vs. 0.26 µM from competitive FP). Obviously, more residues located N-terminally of the LXXLL motif in STAT6, play an important role in stabilizing the protein binding to NCoA1^9^. Yet, the molecular recognition mechanism of NCoA1 by the STAT6 transactivation domain is still poorly understood. Here, we report the structural characterization of the complex between a STAT6-derived peptide encompassing the region from Gly^783^ to Gly^814^ and the NCoA1 PAS-B domain^257–385^ using NMR and X-ray crystallography. The structural characterization of the NCoA1^257–385^/STAT6^783–814^ complex demonstrates that the STAT6^783–814^ peptide binds to the NCoA1 PAS-B domain^257–385^ by additional amino acids from its N-terminal region resulting in a more extended binding interface with NCoA1 compared to that identified before in the crystal structure with the STAT6^794–814^ peptide^8^. Overall, the data indicate that the conformational propensity of free STAT6^783–814^ peptide in solution on the level of secondary and tertiary structure supports conformational selection as the key mechanism driving the molecular recognition of the coactivator by STAT6.

## Results

### Structural characterization of the STAT6^783–814^ peptide

To gain insight into the structural features of the protein-protein recognition mechanism between STAT6^783–814^ and the NCoA1 PAS-B domain we first investigated by NMR the STAT6^783–814^ peptide in the free form. The ^1^H,^15^N-HSQC spectrum of ^15^N-^13^C-labeled STAT6^783–814^ (SI Fig. 1) shows narrow dispersion of signals in both proton and nitrogen dimensions indicating that the peptide adopts an unstructured conformation in absence of the binding partner. This finding was further supported by the analysis of the 3D ^1^H,^15^N-NOESY-HSQC spectrum that does not show any medium and long range NOEs (SI Fig. 2). To better understand the structural details of the conformational ensemble sampled in solution by the STAT6^783–814^ peptide we also analyzed the backbone chemical shifts that are sensitive reporters of the secondary structure content^10^. Hence, we obtained the complete assignment of proton, Cα and Cβ chemical shifts of STAT6^783–814^ in the free state (see Materials and Methods section) (Table SI 1a).

To obtain secondary structure propensities through the chemical shifts, we used the random coil values suitable for IDPs^11,12^. Overall, the secondary Cα and Hα chemical shifts are relatively small and no patterns could be discerned, consistent with a lack of ordered secondary structure (Fig. 2A,B).

Nevertheless, the positive Cα and negative Hα secondary chemical shifts suggest the presence of a significant amount of transiently formed helix for the residues located in the region containing the LXXLL motif (Glu^799^-Glu^808^) (Fig. 2A,B). Additionally, ^3^JH_N_Hα coupling constants and the intensity ratio of αH-HN(i,i + 1)/HN-HN(i,i + 1) NOEs (Fig. 2C) were analyzed. Altogether the data for the residues located at the N-terminal part of the peptide in the region Ile^786^-Ile^790^ deviate from random coil values indicating helical propensity that is lower than the helical propensity for the helix between residues Glu^799^-Glu^808^.

To further investigate the conformational properties for STAT6^783–814^ in terms of helical populations, we utilized the secondary structure propensity (SSP) approach^13^. Our quantitative analysis (Fig. 2C) reveals more than 30% α-helical propensity in the region from Glu^799^ to Glu^808^ containing the LXXLL motif which is in quantitative agreement with the analysis of the coupling constants (Table SI 1b). In the region from Ile^786^ to Ile^790^ of the N-terminal part of the peptide 10% α-helical propensity is predicted from the SSP analysis as well as the ^3^JH_N_Hα coupling constants analysis (Table SI 1b). Taken together the data clearly indicate that the free STAT^783–814^ peptide does not adopt a completely random coil conformation but contains two regions (Ile^786^- Ile^790^, Glu^799^-Glu^808^) with a significant α-helical propensity of around 10% for the first and 30% for the second sequence.

### Structure of the NCoA1^257–385^/ STAT6^783–814^ complex by X-ray crystallography

In order to understand the increase of affinity of the STAT6^783–814^ peptide compared to the STAT6^794–814^ peptide in complex with NCoA1 by a factor of 10 we first solved the crystal structure of the NCoA-1 PAS-B domain in complex with the STAT6^783–814^ peptide (PDB ID: 5NWX) (SI Tables 2 and 3). In this crystal structure the residues located in the N-terminal part (Gly^783^ - Pro^793^) are not ordered indicating that this region is too dynamic to be resolved in the crystal (SI Fig. 3). Nevertheless, the new X-ray structure of the complex clarifies the structural role of Leu^794^ whose side chain fits into a deep pocket on the cofactor surface formed by Phe^314^, Phe^300^ and Ala^310^. Despite the fact that this amino acid was present in the construct crystallized previously^8^ it was not visible in the X-ray structure. Yet, there is no interaction visible between the residues N-terminal of Leu^794^ in the crystal structure. Therefore, we decided to perform a structural characterization of the NCoA1^257–385^/STAT6^783–814^ complex by NMR.

### Mapping of the NCoA1 PAS-B domain binding site on STAT6^783–814^

**Figure 3:**
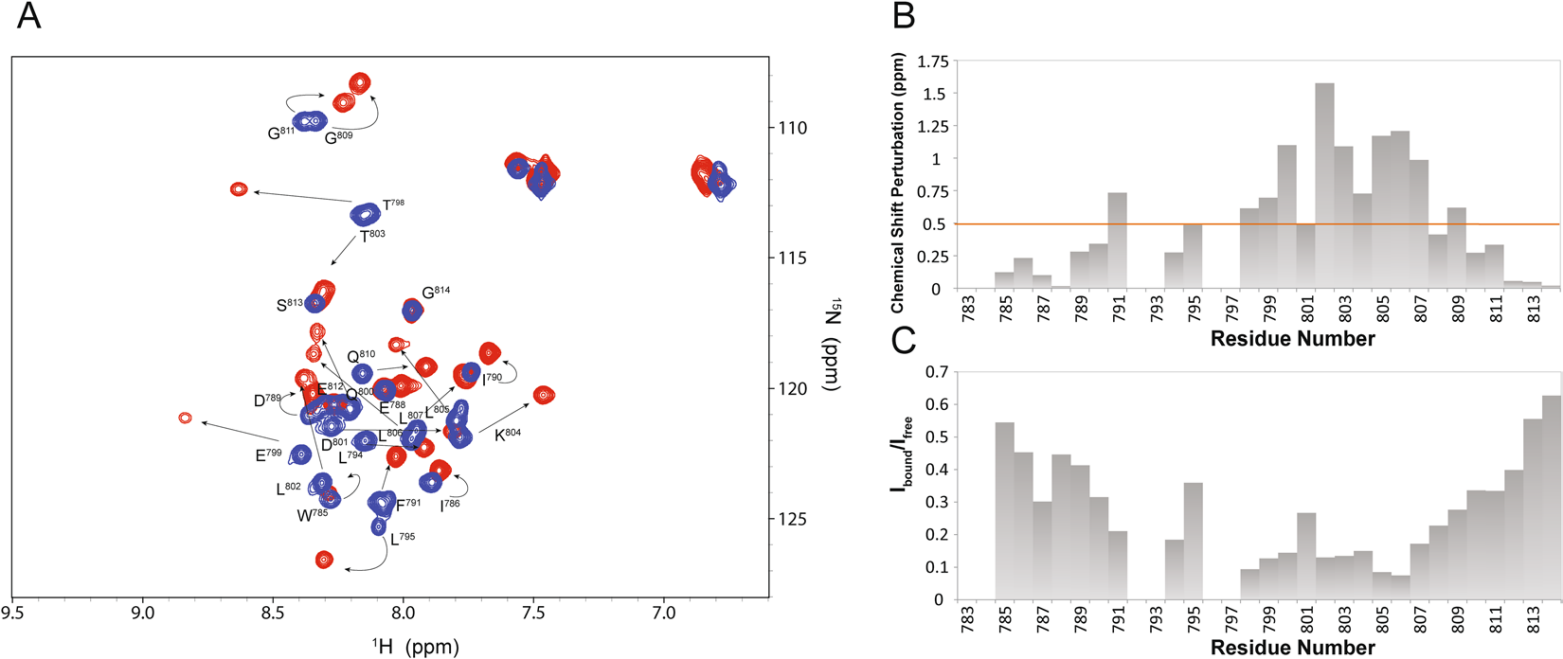
NMR analysis of STAT6^783–814^ binding to NCoA1 PAS-B domain. (**A**) Overlay of the ^1^H,^15^N-HSQC spectra of STAT6^783–814^ in free form (blue) and in complex with the NCoA1 PAS-B domain (red). (**B**) Chemical shift perturbations (ppm) of STAT6^783–814^ upon binding plotted versus the primary sequence; the orange line indicates the mean value. (**C**) Signal intensity ratios of STAT6^783–814^ bound to NCoA1 PAS-B domain (I_bound_) and free (I_free_).

The STAT6^783–814^-NCoA1 PAS-B interaction was first described by investigating the structural changes of the STAT6^783–814^ peptide upon binding with the coactivator. Therefore, the interaction was monitored by acquiring the ^1^H,^15^N-HSQC spectrum of ^15^N-^13^C-labeled STAT6^783–814^ in complex with the unlabeled PAS-B domain of the coactivator. A subset of STAT6^783–814^ backbone amide resonances becomes well dispersed in the presence of the binding partner (Fig. 3A). These changes are indicative of a well-structured region within the bound STAT6^783–814^ peptide. In particular, the resonance peaks of the residues from Thr^798^ to Gly^811^ next to the LXXLL motif are markedly perturbed when STAT6^783–814^ is bound to the NCoA1 PAS-B domain, and among these, the three leucine residues (Leu^802^, Leu^805^ and Leu^806^) are strongly perturbed upon binding (Fig. 3B,C). In agreement with the X-ray data reported previously^8^, these findings indicate that the recognition mechanism of NCoA1 cofactor by STAT6 is principally mediated by the region containing the LXXLL motif with the three leucine residues playing an important role in the complex formation. In addition, in agreement with the X-ray structure (PDB ID: 5NWX), the residues Leu^794^ and Leu^795^ located at the N-terminal region of the peptide show small but significant chemical shift variations upon binding. Furthermore, Phe^791^ also shows chemical shift changes suggesting that the complex may be further stabilized by additional residues flanking the LXXLL motif.

### Chemical shift assignments and conformational analysis of the NCoA1 PAS-B domain in complex with STAT6^783–814^

**Figure 4:**
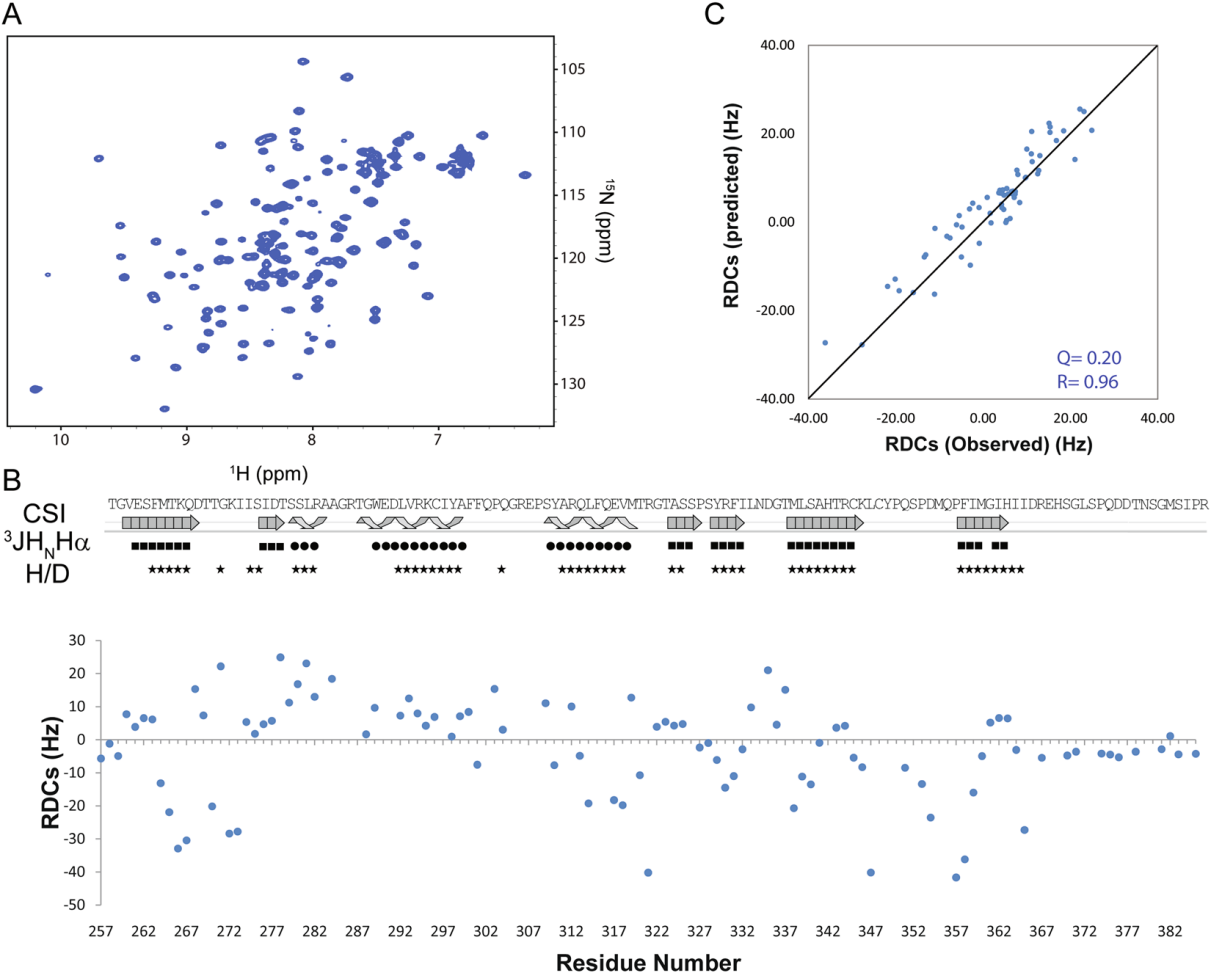
Conformational analysis of the NCoA1 PAS-B domain bound to STAT6^783–814^ by using NMR and X-ray (PDB ID: 5NWX) data. (**A**) ^1^H,^15^N-HSQC spectrum of the ^15^N-^13^C NCoA1^257–385^ in complex with unlabeled STAT6^783–814^. (**B**) RDC values of NCoA1^257–385^ in bound form measured at 900 MHz. A scheme of the secondary structure elements of NCoA1^257–385^ in dependence of the protein sequence shown on top as derived by the Chemical Shift Index (CSI) based on Cα and Hα resonance assignments. ^3^JH_N_Ha coupling constants are also reported and indicated by filled circles and filled squares for values of ^3^JH_N_Hα < 4.5 Hz or > 8 Hz, respectively. The slowly exchanging amide protons are indicated by stars in the H/D line. (**C**) Plot of measured RDCs versus those calculated from the x-ray structure reported in this manuscript (PDB ID: 5NWX) using PALES^49^.

The PAS-B domain of NCoA1 adopts in the complex a stable folded structure (Fig. 4A). A nearly complete ^1^H, ^13^C and ^15^N assignment of the NCoA1^257–385^ domain has been obtained using standard triple resonance experiments (see Materials and Methods). More than 91% of the backbone resonances (^1^H_N_,^15^N,^13^Cα, and ^13^CO) and 89% of all side chain ^13^C and ^1^H resonances were assigned. Secondary structure elements of NCoA1^257–385^ were identified by the analysis of the chemical shift index^14^, and then confirmed by ^3^JH_N_Hα coupling values and hydrogen exchange experiments (Fig. 4B). Specifically, the ^3^JH_N_Hα coupling values drop for the two helices identified before in the free peptide to values indicating close to 100% helix formation. Indeed, as shown in Fig. 4B, NCoA1^257–385^ in complex with the STAT6^783–814^ peptide preserves all secondary structure elements reported in the crystallographic structure of the PAS-B domain in complex with the shorter peptide STAT6^794–814^ (PDB ID: 1OJ5) as well as the structure with STAT6^783–814^ (PDB ID: 5NWX) with the exception of the helix Ile^786^ - Ile^790^ which is fully populated in solution, however, there is no electron density in the crystal structure. To check this result independently and get more insight into the secondary and especially of the tertiary structure adopted by the NCoA1 PAS-B domain in complex with the STAT6^783–814^ we analyzed RDCs that are a sensitive probe of local structure as well as of protein motions^15,16^. In particular, we used the ^1^D_NH_ RDCs to detect if the PAS-B domain of the coactivator undergoes local structural variations, in order to bind with high affinity the STAT6^783–814^ peptide. Weak alignment of the selectively ^15^N-labeled NCoA1^257–385^/STAT6^783–814^ complex was achieved by the addition of filamentous bacteriophage Pf1^17^. Large ^1^D_NH_ RDCs (40 Hz) (Fig. 4B) were obtained for the complex, which indicated substantial alignment and allowed for high sensitivity RDC measurement. In order to understand whether the PAS-B domain bound to STAT6^783–814^ adopts in solution a different conformation with respect to that observed in the crystal of the NCoA^257–385^/ STAT6^794–814^ complex we calculated theoretical RDC values from the X-ray structure reported in this manuscript (PDB ID: 5NWX). Alignment tensors were determined employing a linear fit procedure^18^ using the X-ray structure (PDB ID: 5NWX) and the measured RDCs, but considering only the residues located in the region having a secondary structure in accordance with the chemical shifts, ^3^JH_N_Hα coupling and hydrogen exchange data. Using these alignment tensors together with the crystal coordinates, RDC values were predicted for the entire protein and then compared with the experimental RDC values measured on the NCoA^257–385^/STAT6^783–814^ complex. All RDC values throughout the entire protein, apart from the additional C-terminal tail, which is missing in the crystal structure, are in good agreement with the crystal structure as reflected by the Q factor value (Q = 0.20) and by the Pearson’s correlation coefficient (R = 0.96) (Fig. 4C). This demonstrates that the NCoA1 PAS-B domain in complex with the longer STAT6^783–814^ adopts a conformation similar to the crystal structure (PDB ID: 5NWX) and that any dynamic variations compared to the crystal structure coordinates must be small.

### Identification of the NCoA1 PAS-B/STAT6^783–814^ binding interface

**Figure 5:**
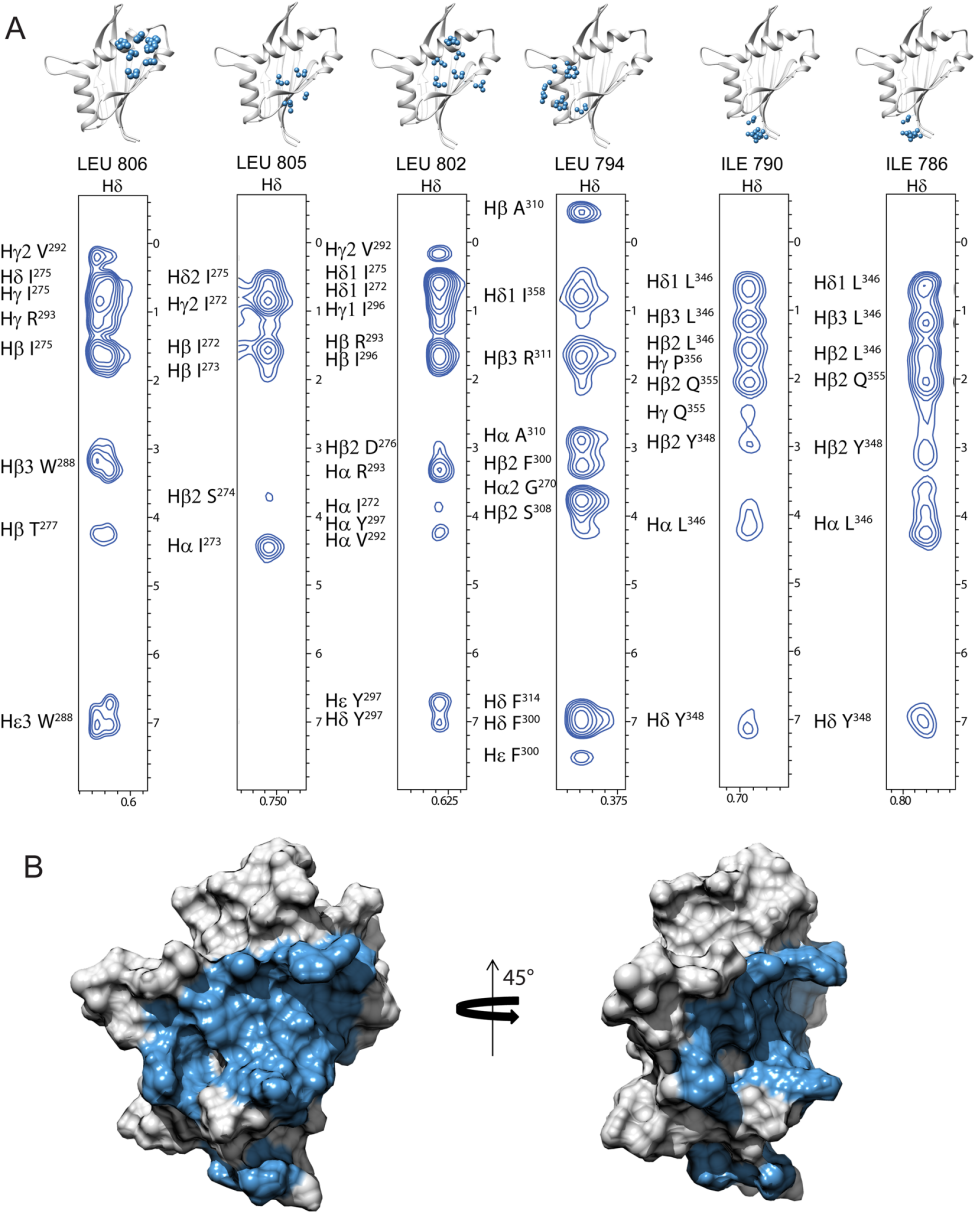
Mapping of the STAT6 binding surface on the NCoA1 PAS-B domain (PDB ID: 1OJ5). (**A**) Strips from the 3D ^13^C-edited/^12^C-filter NOESY experiment measured on the unlabeled NCoA1 PAS-B domain in complex with ^15^N-^13^C STAT6^783–814^ showing inter-molecular NOEs. The strips are related to the residues STAT6 Leu^806^, Leu^805^, Leu^802^, Leu^794^, Ile^790^ and Ile^786^ directly involved in the interaction. (**B**) Mapping of the residues of NCoA1 involved in the interaction onto the x-ray structures (PDB ID: 1OJ5) in two orientations rotated by 45° around the z-axis.

The structural features of the binding mode of the coactivator NCoA1 with STAT6 was determined by intermolecular NOEs measured using an ^13^C-edited/^12^C-filter NOESY-HSQC experiment^19^, exploring the ^15^N-^13^C enriched STAT6^783–814^ peptide and the natural abundance NCoA1 PAS-B domain. Based on the intermolecular NOEs, the interface formed by NCoA1 with STAT6^783–814^ appears to be considerably larger compared to the shorter peptide STAT6^794–814^. A large number of intermolecular NOEs is observed between the side chains of Leu^802^, Leu^805^ and Leu^806^ of STAT6^794–814^ and the NCoA1^257–385^ residues Ile^272^, Ile^273^, Ser^274^, Thr^277^, Trp^288^, Val^292^, Arg^293^ and Tyr^297^ (Fig. 5A). These findings, confirming the crystal structure of the NCoA1^257–385^/STAT6^794–814^ complex, indicate that the three leucine residues of the LXXLL motif play indeed a crucial role in the molecular recognition process. Interestingly, the intermolecular NOE analysis indicates that the NCoA1^257–385^/STAT6^783–814^ complex is stabilized by interactions of additional residues located in the N-terminal region of the peptide. In particular, in agreement with the X-ray structure of the NCoA1^257–385^/STAT6^783–814^ complex the side chain of Leu^794^ shows intermolecular NOEs with the residues Gly^270^, Phe^300^, Ser^308^, Ala^310^, Arg^311^and Ile^358^.

The side chains of Ile^786^ and Ile^790^ that were not observed in the X-ray structure (PDB ID: 5NWX) interact with the NCoA1 residues Leu^346^, Tyr^348^, Gln^355^ and Pro^356^ (Fig. 5A). Notably, the NCoA1 residues showing intermolecular NOEs with the STAT6-derived peptide create a continuous patch on the surface of the PAS-B domain defining an extended binding interface (Fig. 5B). Residues Ile^786^ and Ile^790^ were not present in the peptide crystallized in the complex with the NCoA1 domain^8^ (PDB ID: 1OJ5) and are also not visible in the x-ray structure described above (PDB ID: 5NWX), such that their interaction is only observed in the solution structure and may be responsible for the enhanced binding.

### Structure of the NCoA1^257–385^/STAT6^783–814^ complex by NMR

**Figure 6:**
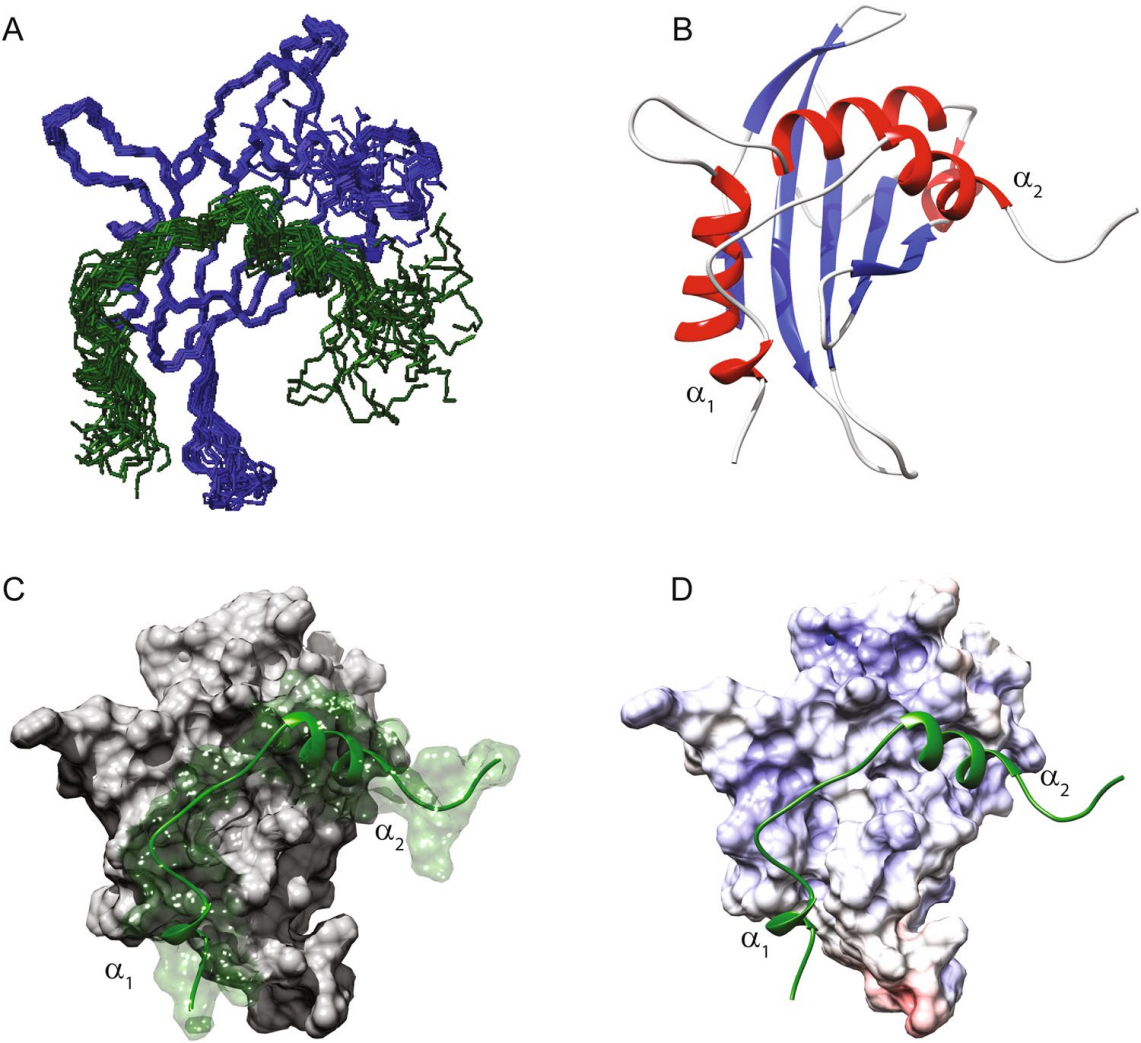
The NMR structure of the NCoA1^257–385^/STAT6^783–814^ complex (PDB ID: 5NWM). (**A**) Overlay of the 20 lowest energy structures of the NCoA1^257–385^/STAT6^783–814^ complex. (**B**) Ribbon drawing of one representative conformer of the NMR structure NCoA1^257–385^/STAT6^783–814^ complex. (**C**) Solvent accessible surface of the NCoA1^257–385^/STAT6^783–814^ complex. The NCoA1 PAS-B domain is depicted in light grey while the STAT6 peptide is reported in dark green. (**D**) Electrostatic surface of NCoA1^257–385^ bound to the STAT6^783–814^ peptide. The positively charged residues are depicted in blue while the negatively charged residues are in red. STAT6^783–814^ is reported in dark green.

The NMR structure of the NCoA1^257–385^/STAT6^783–814^ complex (PDB ID: 5NWM), is of high quality (Table SI 4). In the complex the PAS-B domain adopts a well-defined globular fold (rmsd_BackboneAtoms_^260–367^ = 0.485 Å) (Fig. 6A) ranging from Glu^260^ to Glu^367^ and a dynamically disordered tail at the C-terminus as confirmed by ^15^N-^1^H heteronuclear NOE values (SI Fig. 4). The PAS-B domain shows all structural features known already from the two crystal structures (PDB IDs: 1OJ5, 5NWX) (SI Fig. 5) with a five-stranded anti-parallel β-sheet and three α-helices that connect the second and third β-strand (Fig. 6B).

**Figure 7:**
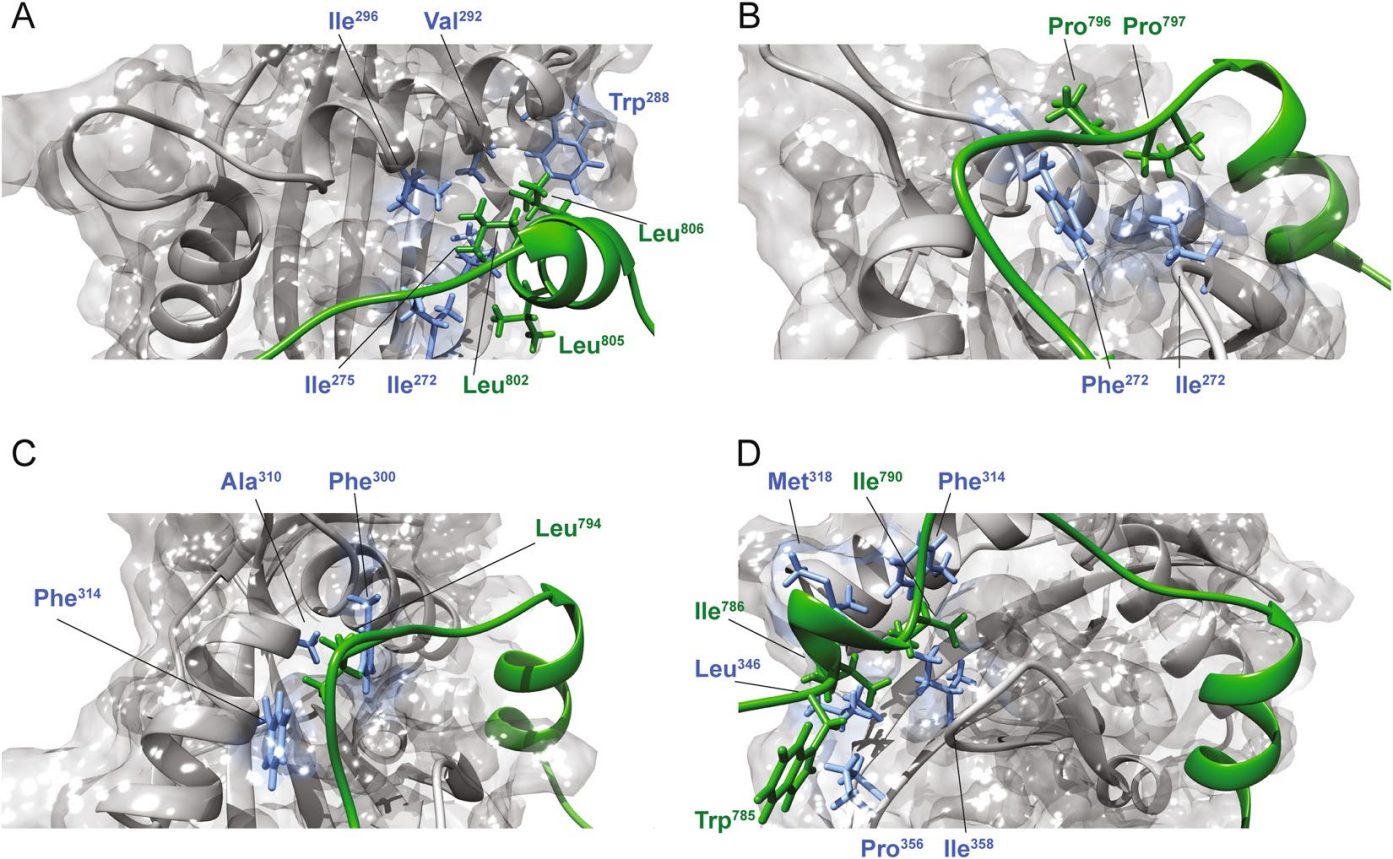
Structural details of the NCoA1^257–385^/STAT6^783–814^ interface as reported by the NMR structure (PDB ID: 5NWM). (**A**) STAT6 Leu^802^, Leu^805^ and Leu^806^ fit into the binding groove of the NCoA1 PAS-B domain. (**B**) STAT6 Pro^796^ and Pro^797^ interact with residues Ile^272^, Ile^296^, Tyr^297^ and Phe^300^ located in the C-terminal part of helix α3 of the NCoA1 PAS-B domain. (**C**) STAT6 Leu^794^ is embedded in a shallow hydrophobic depression constituted by the residues Phe^314^, Phe^300^ and Ala^310^ of the NCoA1 PAS-B domain. Ile^790^ is inside a less deep hydrophobic pocket formed by Phe^314^, Met^318^ and Ile^358^. (**D**) STAT6 Ile^786^ interacts with Met^318^, Leu^346^, Pro^356^ and Ile^358^ of the NCoA1 PAS-B domain.

The STAT6^783–814^ peptide in complex with the NCoA1 PAS-B domain presents a short flexible N-terminal tail with a one-turn α-helix (α1) in the region from Ile^786^-Ile^790^ (as confirmed by the low values of the ^3^JH_N_Hα couplings and the NOEs (Fig. 2C) that is connected by a linker that adopts an extended conformation to the second α-helix (α2) formed by the residues from Glu^799^ to Glu^808^. The STAT6 peptide binds into a shallow groove at the surface of the NCoA1 PAS-B domain, (Fig. 6C,D) with the specific contacts already known from the crystal structures (SI Fig. 5). Shortly, Leu^802^, Leu^805^ and Leu^806^ form the major hydrophobic side chain contacts (Fig. 7A) while Pro^796^ and Pro^797^ make contact with the residues Ile^272^ and Phe^300^ located in the C-terminal part of helix α3 of the PAS-B domain (Fig. 7B). These findings are also in line with the alanine scanning mutagenesis data of Robinson and coworkers^9^. They found that the L802A, L806A and P797A single mutants abolished the NCoA1^257–385^/STAT6^794–814^ complex formation in their FP assay and that the L805A mutant reduced the affinity considerably.

Importantly, the interaction between the NCoA1 PAS-B domain and STAT6^783–814^ is further stabilized by the interaction of STAT6 Leu^794^ with a shallow hydrophobic indentation constituted by the PAS-B domain residues Phe^314^, Phe^300^ and Ala^310^ (Fig. 7C), seen also in the X-ray structure and in agreement with the alanine scanning mutagenesis data where F794A reduced the affinity about 50-fold^9^. In addition, the two isoleucine residues (Ile^786^, Ile^790^) located in the N-terminal part of the STAT6 peptide bind into a shallow hydrophobic pocket on the surface of the PAS-B domain formed by Phe^314^, Met^318^, Leu^346^, Pro^356^ and Ile^358^ (Fig. 7D). Moreover, also STAT6 Trp^785^ slightly contributes to stabilize this interaction making contacts with Pro^356^ of the PAS-B domain. Altogether, the NMR structure indicates that the coactivator recognition mechanism by STAT6 occurs by the formation of a complex in which two folded regions are connected by a linker that adopts an extended conformation. This backbone conformation is further stabilized due to the side chain interaction of Leu^794^ with NCoA1. Thus despite the invisibility of the peptide N-terminal to Leu^794^ in the X-ray structure, the complex formation between the NCoA1 PAS-B domain and STAT6^783–814^ is well defined by specific interactions in solution. This is in contrast to a fuzzy complex^20,21^ as it has been identified e.g. for the interaction between the transcription activator GCN4 and a subunit of the Mediator complex^22^.

Last, in both X-ray structures as well as in the NMR structure the C-terminal region of the STAT6 peptide downstream of Glu^808^ is disordered, in line with the FP assay data with C-terminally truncated peptides that still bound with high affinity to the NCoA1 PAS-B domain^9^.

### Tertiary structural preorganization of free STAT6^783–814^ before forming the NCoA1^257–385^/STAT6^783–814^ complex

We then set out to investigate whether in addition to the preformation of secondary structure elements, there is also a preformation of tertiary structure, i.e. a preorganization of the relative orientation of the two α-helices in the free STAT6 peptide, to facilitate binding to NCoA1^257–385^. Therefore, we evaluated the RDCs measured for STAT6^783–814^ in the free (Table SI 5) and bound forms and compared them to the values predicted from the NMR structure of the complex (PDB ID: 5NWM). Remotely related are earlier studies where proteins were unfolded in urea or guanidinium hydrochloride and the RDCs were compared with the RDCs from the folded forms^23,24^ and the 3D topology was retained in part. Indeed, RDCs are faithful reporters on the relative orientation of structural segments to each other. For the bound form we find a Q factor of 0.17 which is slightly higher than for the PAS-B domain (Q = 0.14) but, as reported by the normal scalar product NSP, with a similar alignment tensor (Table SI 6). Accordingly, fitting of the experimental RDCs of STAT6^783–814^ bound to PAS-B by using the tensor derived from the RDCs of the PAS-B domain resulted in a Q factor of 0.23 (Table SI 6). Taking now the experimental RDCs of the free form (ranging from 1 to 10 Hz) (Table SI 5), the Q factor increased only to 0.32. In addition, for several structural models (SI Fig. 5) in which the N-terminal helix was rotated by 30° about any axis perpendicular to the axis of the C-terminal helix, the Q factor increased to values between 0.36 and 0.54 (Table SI 7). Finally, in order to consider in our analysis the conformational heterogenety of the STAT6^783–814^ in the free form, we generated, as reported in the materials and methods and illustrated in the supplementary information (SI Fig. 7A–C), a pool of random conformers to properly describe the conformational space sampled by the peptide in solution. Interestingly, for all models of the conformational ensemble (SI Fig. 8A), we find Q factors higher than 0.32 for fitting the RDCs of the free form of STAT6^783–814^ (SI Fig. 8B).

Even in this much more exhaustive ensemble, we find a Q factor close to 0.32 only for two structural models, namely SM20 and SM21. For these models the angle between the α-helices (interhelical angle θ) is θ_SM20_ = 51°, θ_SM21_ = 89° compared to θ_NMR_ = 134°) (SI Fig. 8C) which will be further discussed below (see Discussion).

These findings indicate that indeed the tertiary structural elements are preorganized in the free form to facilitate binding. This finding differs from the mentioned studies^23,24^ in the sense that the “unfolding conditions” for STAT6^783–814^ are constituted by the absence of the binding partner PAS-B and don’t require chemical denaturants.

## Discussion

How molecular recognition and binding occurs between highly flexible protein domains, is not yet well understood. The conformational selection theory provides a very elegant explanation for molecular recognition, especially in the context of partially structured protein regions^25–28^.

A detailed understanding of the fundamental mechanisms of the molecular recognition of the coactivator NCoA1 by STAT6 is central to understanding biology at the molecular level of this interaction. The NMR structure of the NCoA1^257–385^/STAT6^783–814^ complex indicates that the coactivator recognition mechanism by STAT6 occurs by the formation of a partially ordered complex in which two α-helical regions (Ile^786^-Ile^790^ and Glu^799^-Glu^808^) are connected by a linker that adopts an extended, albeit dynamic, conformation. This dynamics in the linker may act more strongly onto the N-terminal region of the STAT6^783–814^ peptide that has fewer interactions with the NcoA1 PAS-B domain than the C-terminal region, ultimately rendering residues 783–793 disordered in the X-ray structure.

As mentioned above, the conformational characterization of STAT6^783–814^ in the free form indicates that the peptide is prestructured in the two regions Ile^786^-Ile^790^ and Glu^799^-Glu^808^ with a significant propensity to occupy α-helical conformation, 10% and 30%, respectively. Regarding the STAT6^783–814^ peptide in the bound form, the analysis from chemical shifts, ^3^JH_N_Hα couplings and the relative weight of interresidual NOEs (Fig. 2C) suggests that the STAT6^783–814^ peptide presents a flexible N-terminal tail, followed by an α-helix in the region Ile^786^-Ile^790^ whose population increases from around 10% in the free form to close to 100% upon binding to NcoA1 PAS-B (Fig. 2C) (SI Fig. 9). Then a short extended linker follows and finally the second α-helix (Glu^799^-Glu^808^) (Fig. 2A,B) whose population increases from 30% to close to 100%. Given that existing prestructured motifs are enhanced in the bound form, the data suggest that the recognition of the coactivator by STAT6 occurs by a conformational selection^25^ mechanism regarding the secondary structure. We then investigated whether there is also preorganization of the binding motif, i.e. the two helices on the tertiary structural level and fitted experimental RDCs of the free and bound forms to the bound structure and a distorted bound structure. We find indeed that the experimental RDCs of the free peptide fit well to the bound structure and that a rigid body rotation of the N-terminal helix away from this orientation as well as the use of additional conformers with larger conformational heterogenety, deteriorates the fit. These findings indicate that not only on the secondary but also on the tertiary level, i.e. the arrangement of the two helices, there are prestructure motifs which are then also found in the bound form.

**Figure 8:**
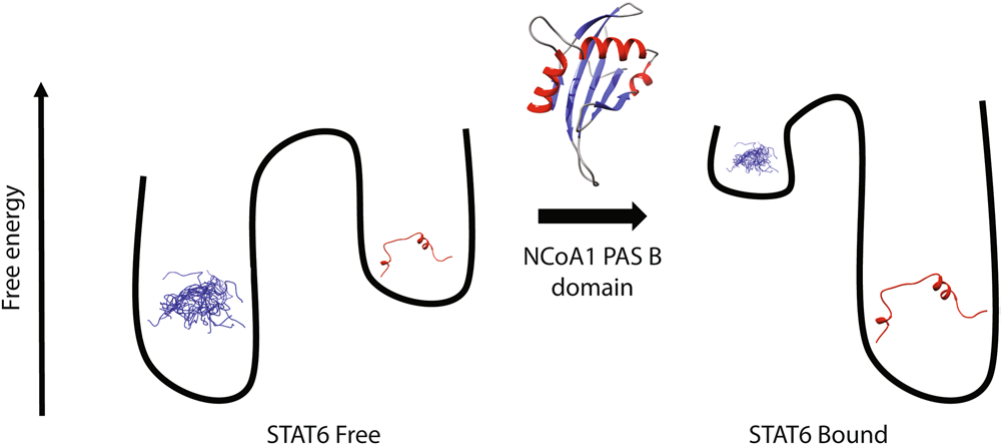
Molecular recognition mechanism. Cartoon representation of the conformational selection mechanism. The binding of the coactivator changes the free-energy landscape of STAT6^783–814^.

Overall the data supports conformational selection^25–28^ in this region as the key mechanism driving the molecular recognition of the NCoA1 PAS-B domain by STAT6 in which the coactivator strongly shifts the α-helical propensity in the regions Ile^786^-Ile^790^ and Glu^799^-Glu^808^ to an even more populated helical state (Fig. 8). In particular, the two helices are present at 10% and 30% population in the free form and become fully populated in the bound form making conformational selection the probable binding mechanism. The 3D arrangement of the two helices found in the bound form is also very likely to prevail in the free form based on RDCs. This suggests a conformational selection not only for the secondary but also for the tertiary structure. This similarity of 3D arrangement of secondary structures in the free and bound form was further investigated by exhaustive sampling of the conformational space. In 77 clusters that deviated sufficiently from the bound structure we found only two that had a similar quality factor for the RDCs. This is still a very strong support for the similarity of the 3D arrangement in the free and bound structure for the following reason: It should be noted that the binding peptide comprises two helices in which the NH vectors point along the helix axis and indeed there is little variation of the respective RDCs (+6 Hz for the C-terminal helix and around −1 Hz for the N-terminal helix). In addition, the peptide contains an extended stretch (F^791^, L^794^, and L^795^) where also little variation of the dipolar couplings is observed (around −9 Hz). For an accurate tensor determination one needs normally at least 5 independent orientations. Thus, it is not surprising that there is symmetry related degeneracies of possible orientations of the helices which fit to the RDCs. Even if these additional structures were populated, the conformational space would still be restricted to an ensemble with less members than a totally disordered peptide, supporting the mechanistic conclusion that a preformation of the bound 3D conformation happens in the free form.

Conformational selection has been observed for quite a number of interactions between structurally well ordered domains and disordered transactivation domains containing an amphipathic α-helical binding motif (ΦXXΦΦ, Φ being a bulky hydrophobic residue) of transcription factors, e.g. the interaction of the transactivation domain of c-Myb with the KIX domain of CBP/p300^29^. In this complex as in other such complexes additional interactions outside the core binding motif that confer specificity have been structurally resolved. But to our knowledge the combination of a highly ordered amphipathic α-helical binding motif interaction with another N-terminally located second α-helical binding motif of equally high specificity in combination with a prearranged 3D arrangement of these helices is so far uniquely observed in the NCoA1 PAS-B/STAT6 transactivation domain complex described in this work and likely explains the higher affinity of STAT6^783–814^ compared to STAT6^794–814^ to the NcoA1 PAS-B domain.

## Materials and Methods

### Protein expression and purification

The recombinant NCoA-1 PAS-B domain comprising amino acids 257–385 of NCoA-1 was expressed and purified as previously published^8^. The fragment containing amino acids 783–814 of STAT6 was produced recombinantly as an N-terminal Z-tag fusion protein with a His_7_-tag and a TEV cleavage site between Z-tag and the target sequence. After TEV cleavage and removal of the Z-tag by Ni-NTA resin (Qiagen) the STAT6 peptide was further purified by reversed phase HPLC. Expression of labeled protein was performed in Toronto minimal medium with ^15^N ammonium chloride as nitrogen source and U-^13^C_6_-D-glucose as carbon source. The STAT6 peptide was added in 1.5 fold molar excess and the complex of the PAS-B domain with this peptide was purified by gelfiltration on a Superdex 75 column (GE Healthcare).

### NMR spectroscopy

NMR samples were made up in the following buffer: 50 mM HEPES, pH 7.0, 150 mM NaCl, 2 mM DTT and 10% ^2^H_2_O. Complex samples were made up by adding STAT6 peptide in 1.5 fold molar excess. All spectra were recorded at a total protein concentration of 1 mM and were carried out at 309 K on Bruker 600, 700, 800, 900 NMR spectrometers equipped with a cryogenic probe and on Bruker 600, 700 NMR spectrometers equipped with a triple resonance probe head. The following experiments were recorded:

- on samples of ^15^N- or ^15^N-^13^C-labeled STAT6^783–814^ in the free form ^1^H,^15^N-HSQC, ^1^H,^13^C-HSQC, HNHA, HNCA, HNCACB, CBCACONH, ^1^H,^15^N NOESY-HSQC and ^1^H,^15^N TOCSY-HSQC^30^.

-on sample of ^15^N-^13^C-labeled STAT6^783–814^ in complex with unlabeled NCoA1^257–385 1^H,^15^N-HSQC, ^1^H,^13^C-HSQC aliphatic, ^1^H,^13^C-HSQC aromatic, HNHA, HNCA, HNCACB, CBCACONH, ^1^H,^15^N NOESY-HSQC, ^1^H,^15^N TOCSY-HSQC, HCCH-TOCSY, ^1^H,^13^C NOESY-HSQC aliphatic, ^1^H,^13^C NOESY-HSQC aromatic^30^, ^1^H,^13^C-edited/^12^C-filter NOESY-HSQC^19^.

-on sample of ^15^N-^13^C-labeled NCoA1^257–385^ in complex with unlabeled STAT6^783–814 1^H,^15^N-HSQC, ^1^H,^13^C-HSQC aliphatic, ^1^H,^13^C-HSQC aromatic, HNHA, HNCA, HNCACB, CBCACONH, ^1^H,^15^N NOESY-HSQC, ^1^H,^15^N TOCSY-HSQC. HCCH-TOCSY, ^1^H,^13^C NOESY-HSQC aliphatic, ^1^H,^13^C NOESY-HSQC aromatic.

The ^15^N edited NOESY-HSQC and ^13^C edited NOESY-HSQC experiments were acquired with a mixing time of 100 ms and 80 ms, respectively.

Slowly exchanging amide protons were identified in an ^1^H,^15^N-HSQC spectrum reordered immediately after exchanging the proton into a buffer prepared with ^2^H_2_O.

Vicinal (three-bond) HN-Hα coupling constants (^3^JH_N_Hα) were evaluate from cross-peak intensities in quantitative J-correlation (HNHA) spectra^31^. Backbone torsion angles were estimated from Cα, CO, Cβ, N, HN and Hα chemical shift using the program TALOS+^32^.

Residual dipolar couplings ^1^D_NH_ RDCs for the ^15^N-labeled STAT6^783–814^/U-NCoA1^257–385^ and U-STAT6^783–814^/ ^15^N-labeled NCoA1^257–385^were measured by taking the difference in the one-bond ^1^H-^15^N splittings (^1^J_NH_+ ^1^D_NH_) in aligned (~20 mg/ml phage pf1^17^) and isotropic media using an in-phase/anti-phase (IPAP) HSQC experiment^33^.

The observed chemical shift change (Δδ_obs_) for each backbone amide between the STAT6^783–814^ in the free and bound form was measured as the weighted average of the proton and nitrogen chemical shift changes by using equation (1)^34^:

1 $${{\rm{\Delta }}\delta }_{{\rm{o}}{\rm{b}}{\rm{s}}}={[({{{\rm{\Delta }}\delta }^{2}}_{{\rm{H}}{\rm{N}}}+{{{\rm{\Delta }}\delta }^{2}}_{{\rm{N}}}/25)/2]}^{1/2}$$

For the evaluation of the ^15^N-[^1^H] steady-state heteronuclear NOE two ^1^H,^15^N-HSQC were acquired (in one the protons were unsaturated and in the other the protons were saturated for 3 s).

For the secondary structure analysis and the secondary structure propensity the random coil chemical shifts of Zhang *et al*.^12^ were used. The SSP scores were calculated with the random chemical shifts and the average secondary shifts for fully formed secondary structure^12^ as described previously^13^.

The evaluation of the alignment tensors was performed by using the normalized scalar product (NSP) defined by equation (2):

2 $$NSP=\frac{\langle {{\rm{S}}}^{sample1}{|S}^{sample2}\rangle }{\sqrt{\langle {{\rm{S}}}^{sample1}{|S}^{sample1}\rangle \langle {{\rm{S}}}^{sample2}{|S}^{sample2}\rangle }}$$

where the scalar product between the two vectors formed from the Saupe matrix elements can be defined according to equation (3):

3 $$\langle {{\rm{S}}}^{sample1}{|S}^{sample2}\rangle =\sum _{\begin{array}{c}i=x,y,z\\ j=x,y,z\end{array}}{S}_{ij}^{sample1}{{\rm{S}}}^{sample2}$$

in which *Sij* are the elements of the 3 × 3 Saupe matrices.

The values of the NSP close to 1.0 indicate that the two alignment tensors differ only by a scaling factor, whereas the values around 0.0 suggest that the alignment frames are orthogonal. The situation where NSP = −1 indicates that the two alignment tensors are antiparallel.

The quality factor (Q) is defined by equation (4)^35^:

4 $$Q=\,\frac{{({\sum }_{i=1}^{N}{({D}_{i}^{exp}-{D}_{i}^{calc})}^{2}/N)}^{1/2}}{{({\sum }_{i=1}^{N}{({D}_{i}^{exp})}^{2}/N)}^{1/2}}$$

The spectra were processed using NMRpipe^36^ and analyzed using SPARKY^37^ and CARA^38^. ^1^H, ^13^C and ^15^N chemical shifts were calibrated indirectly by external DSS references.

### Structure Calculation

NOE-derived distance constraints, coupling constants, TALOS dihedral angles, hydrogen bonds and residual dipolar couplings were used to calculate the structure of the NCoA1^257–385^/ STAT6^783–814^ complex with the program CYANA 3.0^39^. The input data for the final structure are reported in Table SI 4. A total of 100 structures was calculated, and the 20 conformers with the lowest CYANA target function were selected. The small number of residual constraint violations indicates that the input data represent a self-consistent set and that the constraints are well satisfied in the calculated conformers. The structures were visualized and evaluated by using the programs, MOLMOL^40^, CHIMERA^41^, PROCHECK-NMR^42^ and MOLPROBITY^43^. The Adaptive Poisson-Boltzmann Solver (APBS)^44^ was used to calculate spatial distributions of electrostatic potentials using the linearized Poisson-Boltzmann equation and parameters from the PQR files obtained using the PDB2PQR server^45^. The electrostatic map was generated with CHIMERA^41^.

### Generation of the conformational ensemble for RDCs evaluation

A conformational sampling approach was used to generate a conformational ensemble of the free STAT6 in order to describe the conformational space sampled by the peptide in the absence of the binding partner more comprehensively. The Normal Mode-based Simulation (NMSim)^46^ approach has been shown to be a computationally efficient alternative to molecular dynamics simulation for conformational sampling of proteins. Therefore, starting from the NMR structure of the STAT6 peptide in complex with the co-activator, an ensemble of 2500 conformers was generated with the program NMSim^46^ by using the default parameters for large scale motions. Then, the conformational ensemble was clustered by using the software NMRCLUST^47^ implemented in the program CHIMERA^41^. A total of 153 clusters were found and for each of them the representative structure was considered as reference model. Successively, the representative models were further filtered by considering only the conformers in which the θ angle between the first and the second α-helix deviated from this angle in the NMR structure by more than ±30°, i.e. all structures were excluded where this angle was between 104° and 164°. This procedure resulted in the selection of 77 conformers which defined the final conformational ensemble for the evaluation of the RDCs. The structural models (SM1, SM2, SM3, SM4) of the STAT6 peptide obtained from the NMR structure of the complex by rotating the α1 helix by 30° with respect to the α2 helix were also included in the final ensemble.

### Data availability

Coordinates and structural restraints for the NMR structure and the X-ray of NCoA1^257–385^/STAT6^783–814^complex have been deposited in the PDB under the accession number (PDB ID: 5NWM) and (PDB ID: 5NWX), respectively. The chemical shifts have been deposited in the BioMagResBank, accession number (BMRB ID: 34131). The conformational ensemble for RDCs evaluation generated during the current study is available from the corresponding author upon request.

## Electronic supplementary material

Supplementary Information
