# Supplementary material for: Insight into the molecular recognition mechanism of the coactivator NCoA1 by STAT6
Source: Sci Rep. 2017 Dec 4;7:16845. doi: 10.1038/s41598-017-17088-5 (PMC5714956; doi:10.1038/s41598-017-17088-5)
Supplement: Supplementary file 1 — Supplementary Information [file 41598_2017_17088_MOESM1_ESM.pdf]

Supplementary Information

Insight into the molecular recognition mechanism of the coactivator NCoA1 by STAT6

Luigi Russo[1,3], Karin Giller[1], Edith Pfitzner[2,4], Christian Griesinger[1] and Stefan Becker[1*]

[1]Department for NMR based Structural Biology, Max Planck Institute for Biophysical Chemistry, Am Fassberg 11, 37077 Göttingen, Germany,

[2]Friedrich-Schiller-University Jena, Institute of Biochemistry and Biophysics, Philosophenweg 12, 07743 Jena, Germany.

[3]Present address: Department of Environmental, Biological and Pharmaceutical Sciences and Technologies, University of Campania "Luigi Vanvitelli", 81100 Caserta, Italy.

[4]Present address: University of Kassel, Mönchebergstraße 19, 34109 Kassel, Germany

[*]Corresponding author: sabe@nmr.mpibpc.mpg.de

Table SI 1a. ^{1}H, $^{13}C\alpha$ and $^{13}C\beta$ chemical shifts of the unbound STAT6$^{783\text{-}814}$.

	H^N	αH	βH	others	N	Cα	Cβ
Gly^{783}		---	---	---			
Thr^{784}	---	---	---	---		62.12	69.65
Trp^{785}	8.30	4.68	3.30,3.19		124.30	57.32	29.70
Ile^{786}	7.91	3.98	1.68	γCH2 1.33,1.01 γCH3 0.85 δCH3 0.77	123.72	61.17	38.79
Gly^{787}	7.55	3.67			111.59	45.16	
Glu^{788}	8.08	4.20	1.94, 1.83	γCH2 2.26,2.14	120.02	56.62	30.65
Asp^{789}	8.34	4.48	2.61, 2.50		120.90	54.35	41.10
Ile^{790}	7.76	4.00	1.66	γCH2 1.19,1.06γCH3 0.76 δCH3 0.64	119.47	60.90	38.79
Phe^{791}	8.12	4.74	3.10,2.86		124.47	55.24	42.15
Pro^{792}							
Pro^{793}						62.92	31.89
Leu^{794}	8.18	4.20	1.56	γH1.49 δCH3 0.94,0.81	122.05	55.22	42.30
Leu^{795}	8.13	4.43	1.62	γH1.49 δCH3 0.94,0.85	125.37	55.83	41.95
Pro^{796}							
Pro^{797}						63.40	31.87
Thr^{798}	8.15	4.12	4.07	γCH3 1.20	113.39	61.97	69.98
Glu^{799}	8.42	4.20	1.97, 1.89	γCH2 2.27,2.17	122.54	57.11	30.18
Gln^{800}	8.26	4.19	2.00, 1.94	γCH2 2.35,2.25	120.66	56.64	29.64
Asp^{801}	8.29	4.49	2.04, 1.89	γCH2 2.76,2.58	121.48	54.36	40.93
Leu^{802}	8.34	4.22	1.76	γH1.64 δCH3 0.93,0.78	123.66	56.43	41.87
Thr^{803}	8.17	4.14	4.06	γCH3 1.24	113.48	64.11	69.20
Lys^{804}	7.79	4.18	1.87, 1.78	γCH2 1.42, 1.32 δCH21.69 εCH22.95	121.91	57.03	32.64
Leu^{805}	7.80	4.16	1.60	γH1.54 δCH3 0.92, 0.78	121.25	55.61	42.21
Leu^{806}	7.96	4.26	1.62	γH1.56 δCH3 0.91, 0.78	121.62	55.16	41.82
Leu^{807}	7.98	4.27	1.64	γH1.52 δCH3 0.93, 0.80	121.96	55.96	42.40
Glu^{808}	8.21	4.19	2.03, 1.92	γCH2 2.32, 2.18	120.80	57.01	30.19
Gly^{809}	8.35	3.92			109.76	45.49	
Gln^{810}	8.17	4.31	2.06, 1.94	γCH2 2.39, 2.24	119.39	55.85	29.50
Gly^{811}	8.40	3.93			109.77	45.20	
Glu^{812}	8.31	4.30	1.98, 189	γCH2 2.26, 2.20	120.59	56.67	30.35
Ser^{813}	8.36	4.41	3.88		116.76	58.34	64.07
Gly^{814}	7.98	3.75			117.00	46.18	

Table SI 1b. $^{3}J(H_N,H_\alpha)$ **couplings of the unbound and bound with reference values for the random coil conformation[17] STAT6$^{783\text{-}814}$.**

Residue	$^{3}J(H_N,H_\alpha)$ Free	$^{3}J(H_N,H_\alpha)$ Bound	$^{3}J(H_N,H_\alpha)$ reference coil[17]
Gly^{783}			
Thr^{784}			
Trp^{785}	6.84	5.59	6.5
Ile^{786}	7.34	7.16	7.2
Gly^{787}			
Glu^{788}	6.04	4.45	6.2
Asp^{789}	6.30	4.15	6.6
Ile^{790}	7.00	4.42	7.2
Phe^{791}	6.59	5.34	7.1
Pro^{792}			
Pro^{793}			
Leu^{794}	7.14	5.76	6.6
Leu^{795}	6.44	6.00	6.6
Pro^{796}			
Pro^{797}			
Thr^{798}	7.20	6.99	7.5
Glu^{799}	5.86	4.35	6.2
Gln^{800}	5.99	4.14	6.3
Asp^{801}	6.01	4.30	6.6
Leu^{802}	5.98	3.80	6.6
Thr^{803}	6.30	3.36	7.5
Lys^{804}	6.03	3.96	6.6
Leu^{805}	6.02	3.59	6.6
Leu^{806}	6.17	4.48	6.6
Leu^{807}	6.20	4.47	6.6
Glu^{808}	5.91	4.63	6.2
Gly^{809}			
Gln^{810}	6.67	6.52	6.3
Gly^{811}			
Glu^{812}	7.00	6.37	6.2
Ser^{813}	6.88	6.82	6.6
Gly^{814}			

Table SI 2. X-ray data collection statistics.

**Data statistics**	**NCoA1 PAS-B + STAT6 (783-814)-peptide**
Wavelength	0.7 Å
Beamline	SLS-PX2
Detector	PILATUS 6M
Space group	$P6_2$
a	61.63 Å
b	61.63 Å
c	73.28 Å
Resolution[a]	2.51 Å (2.61-2.51 Å)
Reflections measured	112,455
Unique reflections	5,471
Redundancy	20.51 (19.95)
Completeness(%)	99.8 (98.3)
Mean $I/\sigma(I)$	30.26 (6.1)
R_{rim} (%)[b]	2.6 (13.1)

[a]Values in parentheses are outer-resolution shells.

[b] $R_{rim} = \sum_{hkl} [N/(N-1)]^{1/2} \sum_i |I_i(hkl) - \langle I(hkl)\rangle| / \sum_{hkl} \sum_i I_i(hkl)$, where N is the redundancy and $I_i(hkl)$ is the ith observation of reflection hkl and $\langle I(hkl)\rangle$ is the weighted average intensity for all observations i of reflection hkl.

Table SI 3. X-ray structure refinement statistics NCoA1 PAS-B in complex with the STAT6$^{783\text{-}814}$ peptide.

R-factor[a]	18.35%
R_{free}[b]	23.55%
**Root mean square deviations from ideal geometry**	
Bond lengths	0.016 Å
Bond angles	1.95°
No. of protein residues	118
No. of water residues	8
**Ramachandran plot (%)**	
Favoured	98.18
Allowed	0.91
Outliers	0.91

[a] $R = \sum_{hkl} \left\| F_{obs} \right| - \left| F_{calc} \right\| / \sum_{hkl} \left| F_{obs} \right|$, where F_{obs} and F_{calc} are the observed and calculated structure factors, respectively.

[b] R_{free} was determined using 5% of the data[1].

Table SI 4. NMR structural statistics of the NCoA1$^{257-385}$/ STAT6$^{783-814}$ complex.

**NMR constraints**	
Distance constraints	
Total NOE	1510
Intraresidue	505
Interresidue	1005
Sequential ($\lvert i-j \rvert = 1$)	381
Medium range ($\lvert i-j \rvert < 4$)	251
Long range ($\lvert i-j \rvert > 5$)	280
Intermolecular	93
Hydrogen bonds	40
Total TALOS+ dihedral angle restraints	182
Residual dipolar couplings (^{1}H-^{15}N)	72
**Structure Statistics**	
Violations (mean and SD)	
Distance constraints (Å)	0.029 ± 0.006
Dihedral angle constraints (°)	0.405 ± 0.031
Max. distance constraint violation (Å)	0.045
Max. dihedral angle violation (°)	1.3
RDCs Q-factor	
Alignment tensor 1	0.16 ± 0.01
Alignment tensor 2	0.09 ± 0.03
Rms deviations from idealized covalent geometry	
Bond lengths (Å)	0.001
Bond angles (°)	0.2
**Coordinate precision**	
RMSD from mean structure[b] (Å)	
All backbone atoms	0.485
All heavy atoms	1.310
Ramachandran plot statistics[c] (%)	
Most favored regions	90.8
Additional allowed regions	9.2
Generously allowed regions	0
Disallowed regions	0

The 20 conformes with the lowest energies were selected for statistical analysis.

[a]Q-factor= RMS (D^{calc} - D^{obs})/RMS D^{obs}, where D^{calc} and D^{obs} are calculated and observed RDC values, respectively. The alignment tensor 1 and 2 are related to the NcoA1 PAS-B domain and the STAT6$^{783-814}$ peptide in the complex, respectively.

[b]Only the region 260-367 of the NcoA1 PAS-B domain was used for rmsd calculations. The flexible residues 305, 351-354 were excluded from the analysis

[c]Based on PROCHECK-NMR analysis.

Table SI 5. Residual dipolar couplings measured on the unbound STAT6 peptide.

Residue	$^{1}D_{NH}$ RDCs (Hz)
Gly^{783}	-----
Thr^{784}	-----
Trp^{785}	-----
Ile^{786}	-----
Gly^{787}	-3.13
Glu^{788}	-0.24
Asp^{789}	-0.95
Ile^{790}	-----
Phe^{791}	-9.76
Pro^{792}	-----
Pro^{793}	-----
Leu^{794}	-8.22
Leu^{795}	-9.03
Pro^{796}	-----
Pro^{797}	-----
Thr^{798}	-6.35
Glu^{799}	5.06
Gln^{800}	7.70
Asp^{801}	7.03
Leu^{802}	6.85
Thr^{803}	7.45
Lys^{804}	8.74
Leu^{805}	5.43
Leu^{806}	5.90
Leu^{807}	7.11
Glu^{808}	-----
Gly^{809}	4.07
Gln^{810}	0.89
Gly^{811}	-0.71
Glu^{812}	-0.26
Ser^{813}	-0.77
Gly^{814}	-----

Table SI 6. Alignment tensor parameters related to the fitting of the RDCs of the NCoA1$^{257\text{-}385}$ (bound), STAT6$^{783\text{-}814}$(bound) and STAT6$^{783\text{-}814}$(free) onto the NMR structure of the NCoA1$^{257\text{-}385}$/ STAT6$^{783\text{-}814}$ complex.

	NCoA1$^{257\text{-}385}$ (Bound)[a]	STAT6$^{783\text{-}814}$ (Bound)[b]	STAT6$^{783\text{-}814}$ (Bound)[c]	STAT6$^{783\text{-}814}$ (Free)[d]
A_a (10^{-4})	-16.8	-16.8	-13.5	-4.9
A_r (10^{-4})	-6.9	-6.9	-8.7	-2.4
α (°)	184	184	201	186
β (°)	159	159	157	81
γ (°)	211	211	227	281
Q factor	0.14	0.23	0.17	0.32
NSP[e]	1	1	0.99	0.22

[a]Tensor eigenvalues resulting from the fit of experimental RDCs measured from NCoA1$^{257\text{-}385}$ in complex with STAT6$^{783\text{-}814}$ to the NMR structure reported in this manuscript.
[b]Fitting of the RDCs measured for the bound STAT6$^{783\text{-}814}$ to the NMR structure of the complex by using the alignment tensor parameters obtained by the analysis of the RDCs of NCoA1$^{257\text{-}385}$ in the bound form against the NMR structure.
[c]Tensor eigenvalues resulting from the fit of experimental RDCs measured from the STAT6$^{783\text{-}814}$ in complex with NCoA1$^{257\text{-}385}$ to the NMR structure.
[d]Tensor eigenvalues resulting from the fit of experimental RDCs measured from the free STAT6$^{783\text{-}814}$ to the NMR structure of the NCoA1$^{257\text{-}385}$/ STAT6$^{783\text{-}814}$ complex.
[e]The normalized scalar product relative to the NCoA1$^{257\text{-}385}$(bound) RDCs analysis.

Table SI 7. Alignment tensor parameters related to the fitting of the RDCs measured for $STAT6^{783\text{-}814}$ free onto the structural models (SM1,SM2,SM3,SM4) obtained from the NMR structure of the complex by rotating the α1 helix by 30° about Z, -Z (SM1, SM2) and Y,-Y (SM3, SM4) respectively. Z and Y are perpendicular to the axis (X) of the α2 of the peptide (see figure SI 5). The values related to the NMR structure are also reported.

	SM1	SM2	SM3	SM4	NMR[a]
A_a (10^{-4})	-5.0	-5.3	-4.7	-4.1	-4.9
A_r (10^{-4})	-1.8	-2.0	-2.1	-2.4	-2.4
α (°)	203	184	193	193	186
β (°)	87	84	89	73	81
γ (°)	254	257	248	256	281
Q factor	0.39	0.36	0.41	0.54	0.32

Tensor eigenvalues resulting from the fit of experimental RDCs measured from the free $STAT6^{783\text{-}814}$ to the structural models (SM1, SM2, SM3, SM4) and to the NMR structure of the complex.

Protein crystallization, structure solution and refinement

For crystallization of the NCoA-1 PAS-B (257-385)/ STAT6 (783-814) complex the sample was concentrated to 20 mg/ml. Crystals grew at 293 K during one week from 200 nl drops composed of equal volumes of protein solution and mother liquor consisting of 0.2 M sodium acetate, 0.1 M sodium cacodylate, pH 6.5, 30 % (w/v) polyethylene glycol 8000 (condition 28 of Crystal Screen I (Hampton Research). For cryoprotection crystals were soaked for 1 minute in the mother liquor supplemented with 5 % polyethylene glycol 400 and flash-cooled in liquid nitrogen. Data collection was performed at SLS Villigen, Switzerland (beamline

PXII, Pilatus 6M detector[2]. Data were processed with XDS[3] and scaled with SADABS (Bruker AXS, Madison, Wisconsin, USA). Spacegroup determination and statistical analysis was performed with XPREP (Bruker AXS, Madison, Wisconsin, USA). Data statistics are reported in supplemental Table SI 2. The structure was solved by molecular replacement with PHASER[4] using the structure of the NCoA-1 PAS-B domain with a shorter STAT 6 peptide (Protein Data Bank entry 1OJ5)[5] . Refinement was performed with Refmac[6] alternating with manual model building in Coot[7]. Refinement statistics are given in supplemental Table SI 3.

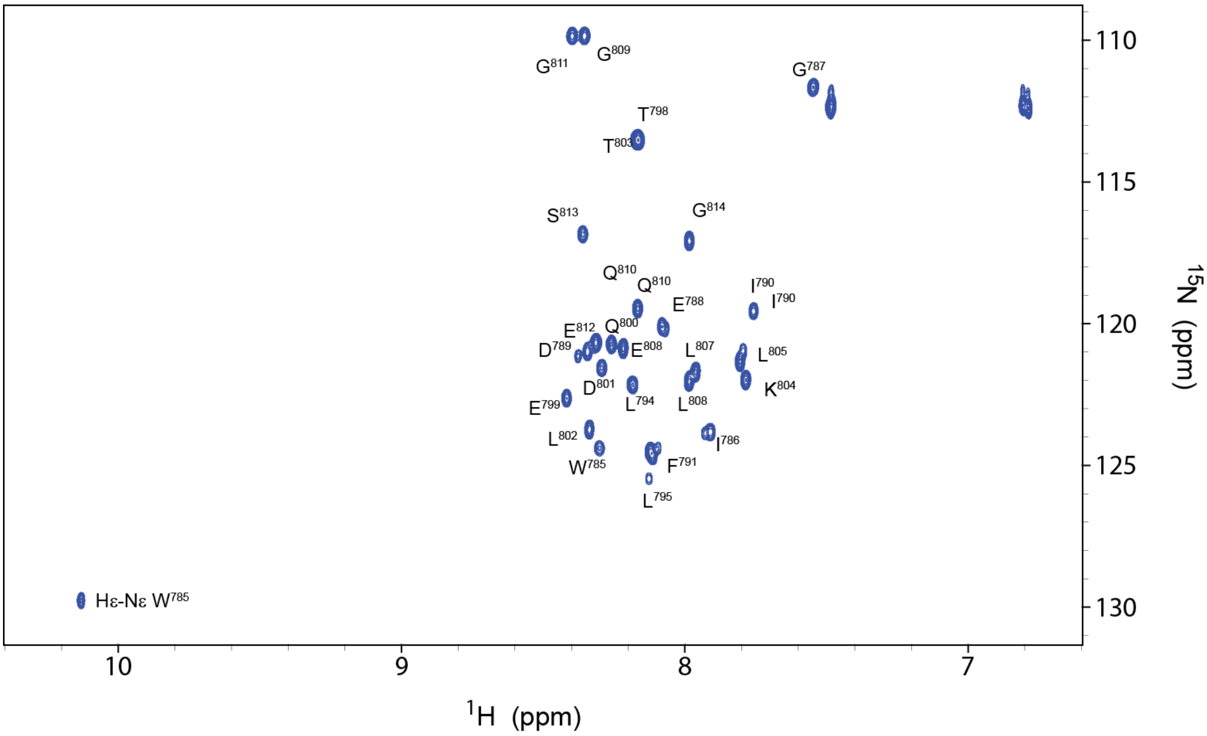

Figure SI 1. The $^{1}H,^{15}N$ heteronuclear single-quantum coherence (HSQC) spectrum of ^{15}N-^{13}C $STAT6^{783\text{-}814}$ in the free form.

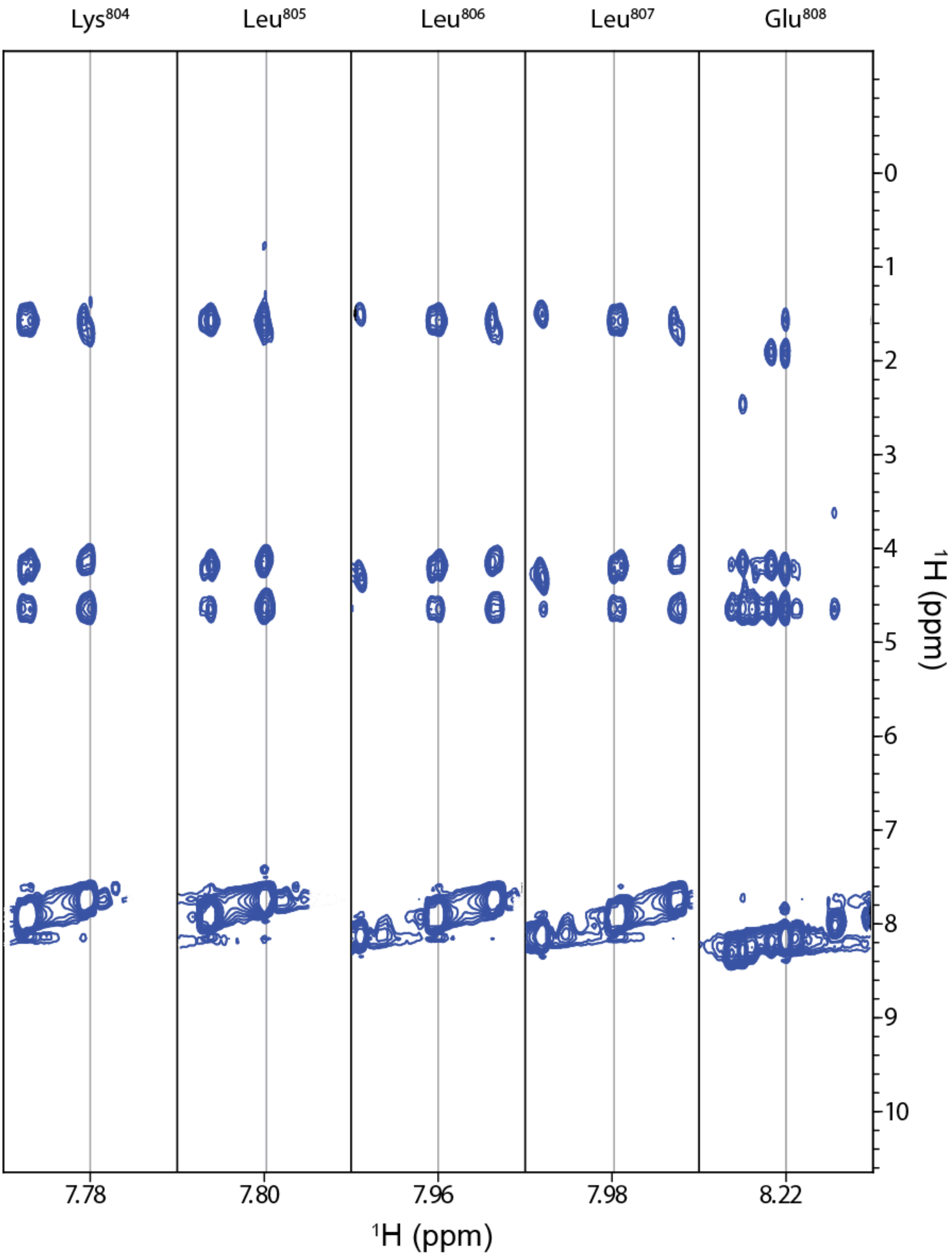

Figure SI 2. Strips from the 3D $^{1}H,^{15}N$ NOESY-HSQC experiment measured on the ^{15}N-^{13}C-labeled STAT6$^{783\text{-}814}$. The strips are related to the residues Lys804, Leu805, Leu806, Leu807 and Glu808 located in the region containing the LXXLL motif.

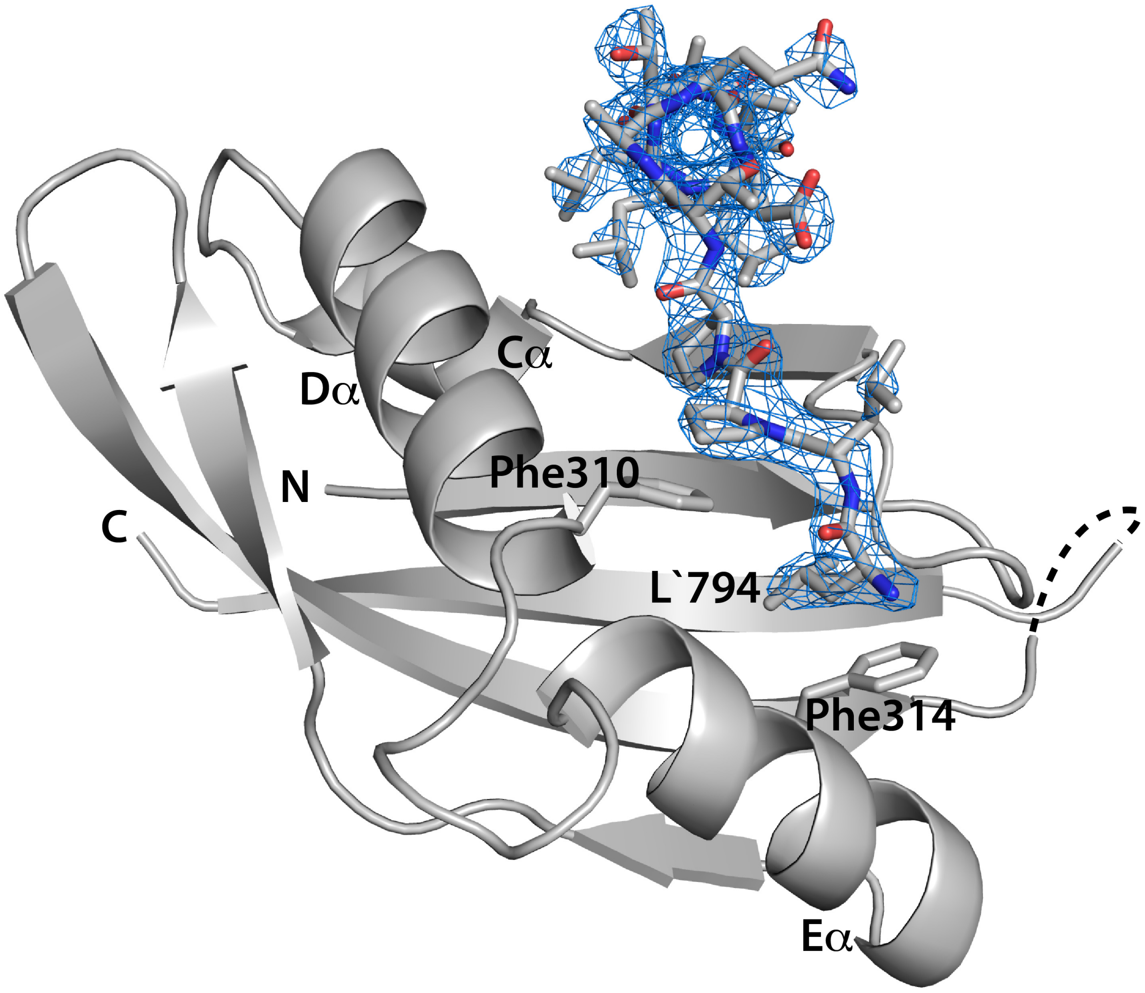

Figure SI 3. Structure of the NCoA1 PAS-B domain in complex with the STAT6 peptide comprising residues 794 – 808.View along the axis of the helix in the STAT6 peptide. The helices of the PAS-B domain are labelled according to[5]. The STAT6 peptide is shown as sticks in the $2mF_o$-DF_c electron density map. The PAS-B domain is shown as a cartoon. Phe^{300} and Phe^{314} are depicted as sticks. They are the major constituents of the hydrophobic pocket where Leu'^{794} of STAT6 is binding.

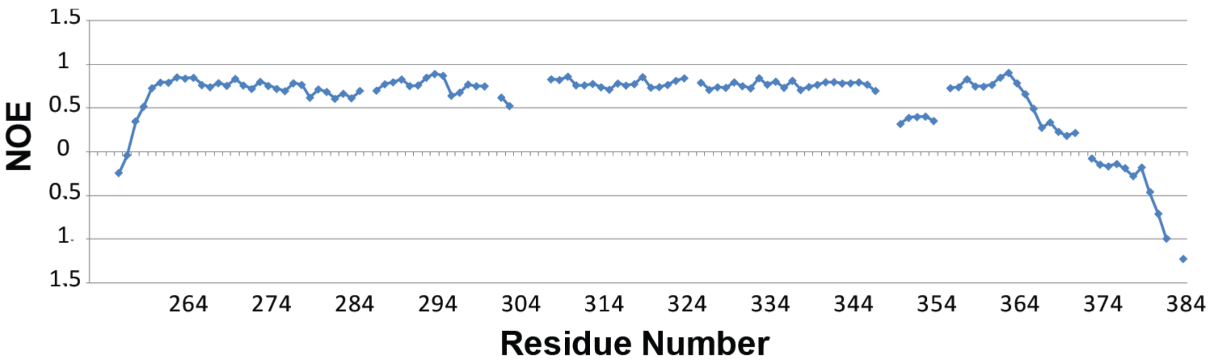

Figure SI 4. ^{15}N-[^{1}H] heteronuclear NOE values of the NCoA1 PAS-B domain.

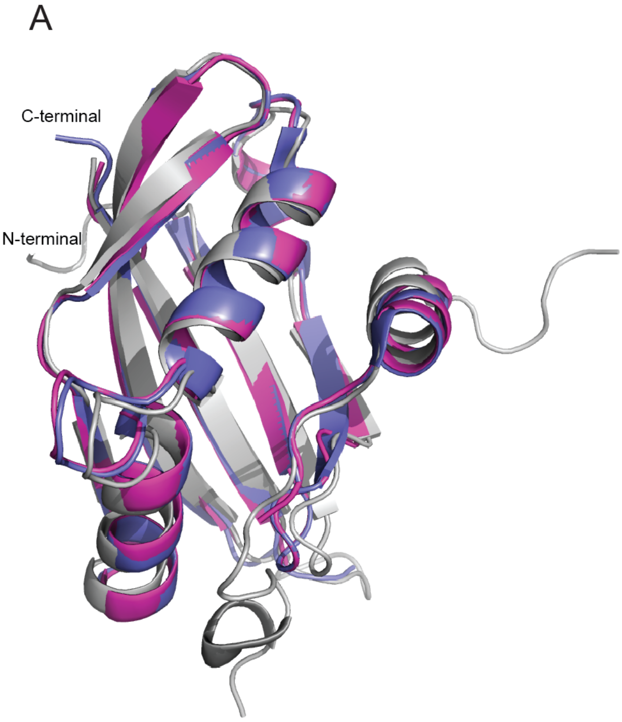

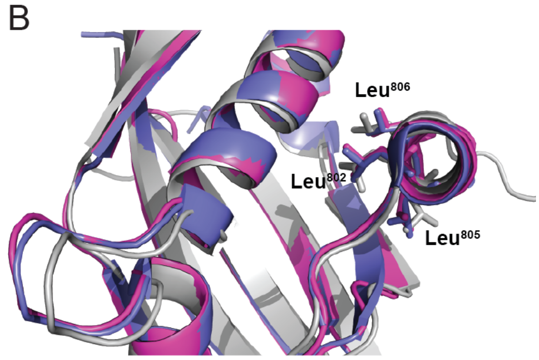

C

PDB ID	5NWM	5NWX	1OJ5
5NWM		0.902	0.854
5NWX	0.902		0.195
1OJ5	0.854	0.195	

Figure SI 5. (A) Comparison of the NMR structure of the NCoA1$^{257\text{-}385}$/ STAT6$^{783\text{-}814}$ complex PDB ID 5NWM (light grey) with both x-ray structures PDB IDs 5NWX (light magenta), 1OJ5 (light violet) after superposition on the backbone atoms of the NCoA1 residues 260-365**.** **(B)** Close-up views of the region Glu^{799} - Glu^{808} of STAT6. The side chains of the STAT6 residues Leu^{802}, Leu^{805} and Leu^{806} are shown as sticks. (C) The root mean square distributions for the backbone heavy atoms of the residues NCoA1 260-365 (RMSD) (Å) between the analyzed structures (PDB IDs 5NWX, 5NWX, 1OJ5).

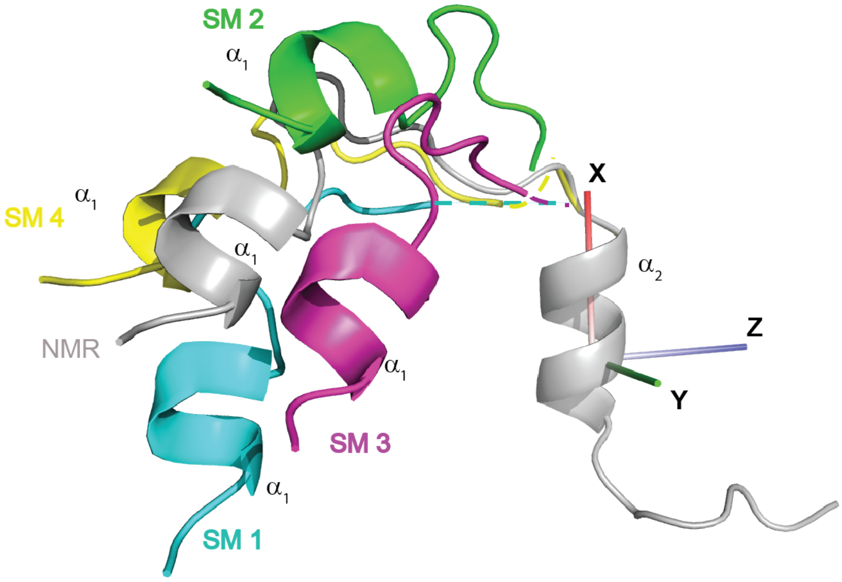

Figure SI 6. Comparison of the structural models SM1 (cyan), SM2 (light green), SM3 (magenta) and SM4 (yellow) obtained from the NMR structure (light grey) of the complex by rotating 30° the α1 about Y,-Y and Z, -Z axis perpendicular to the axis of the α2 of the peptide.

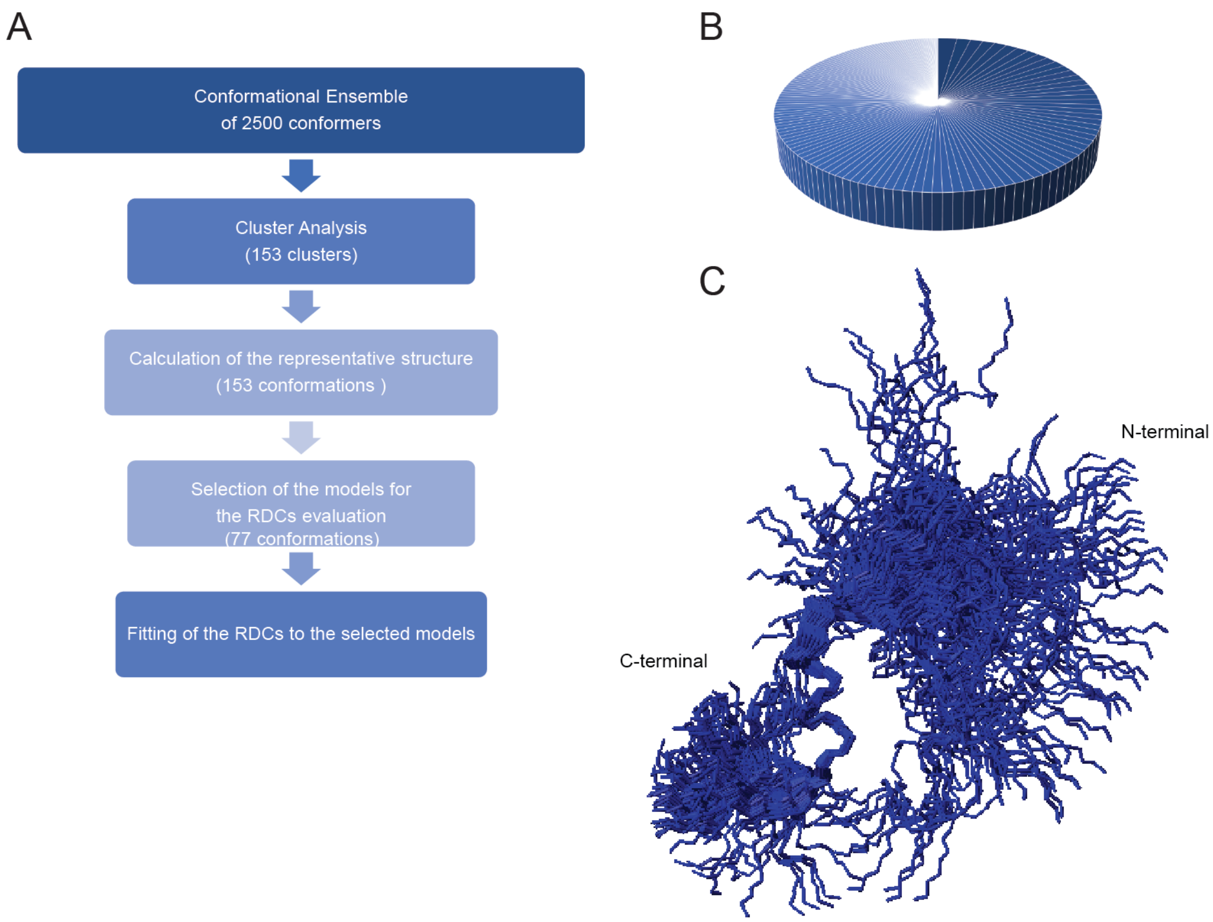

Figure SI 7. (A) Flow-chart of the protocol applied to generate the pool of random conformations for the evaluation of the RDCs measured on the STAT6$^{783\text{-}814}$ free. (B) Cluster analysis of the conformational ensemble. (C) Superposition of the 153 representative models determined for each cluster that were successively selected, as reported in the materials and methods, in 77 conformations for the RDCs evaluation.

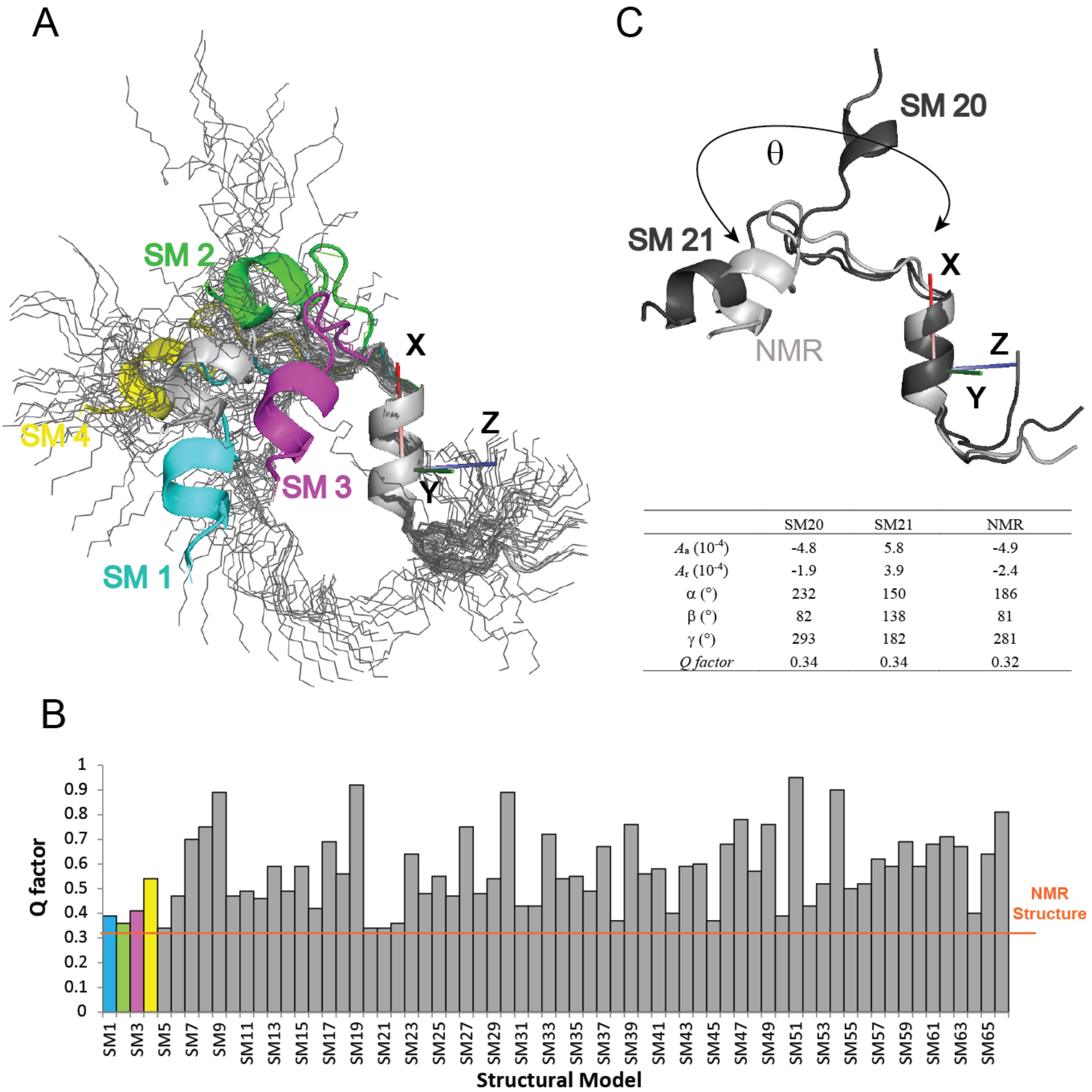

	SM20	SM21	NMR
A_a (10^{-4})	-4.8	5.8	-4.9
A_r (10^{-4})	-1.9	3.9	-2.4
α (°)	232	150	186
β (°)	82	138	81
γ (°)	293	182	281
Q factor	0.34	0.34	0.32

Figure SI 8. (A) Superposition of the structural models used for the evaluation of the RDCs measured for STAT6$^{783\text{-}814}$ free. (B) Comparison of Q factors of the RDCs fitting for the selected structures. The Q factor values for the structural models SM1 (cyana), (light green), SM3 (magenta) and SM4 (yellow) are also reported. The 15 random conformers for which the Q factor is ≥ 1.0 are not included in the plot. The Q factor related to the STAT6$^{783\text{-}814}$ bound conformation (NMR structure) is depicted in orange.(C) Comparison of the structural models SM20 and SM21 with the NMR structure. The θ angle between the first and the second α-helix of the STAT6 peptide is illustrated. Tensor eigenvalues resulting from the fit of experimental RDCs measured from the free STAT6$^{783\text{-}814}$ to the structural models (SM20, SM21) and to the NMR structure of the complex are also reported.

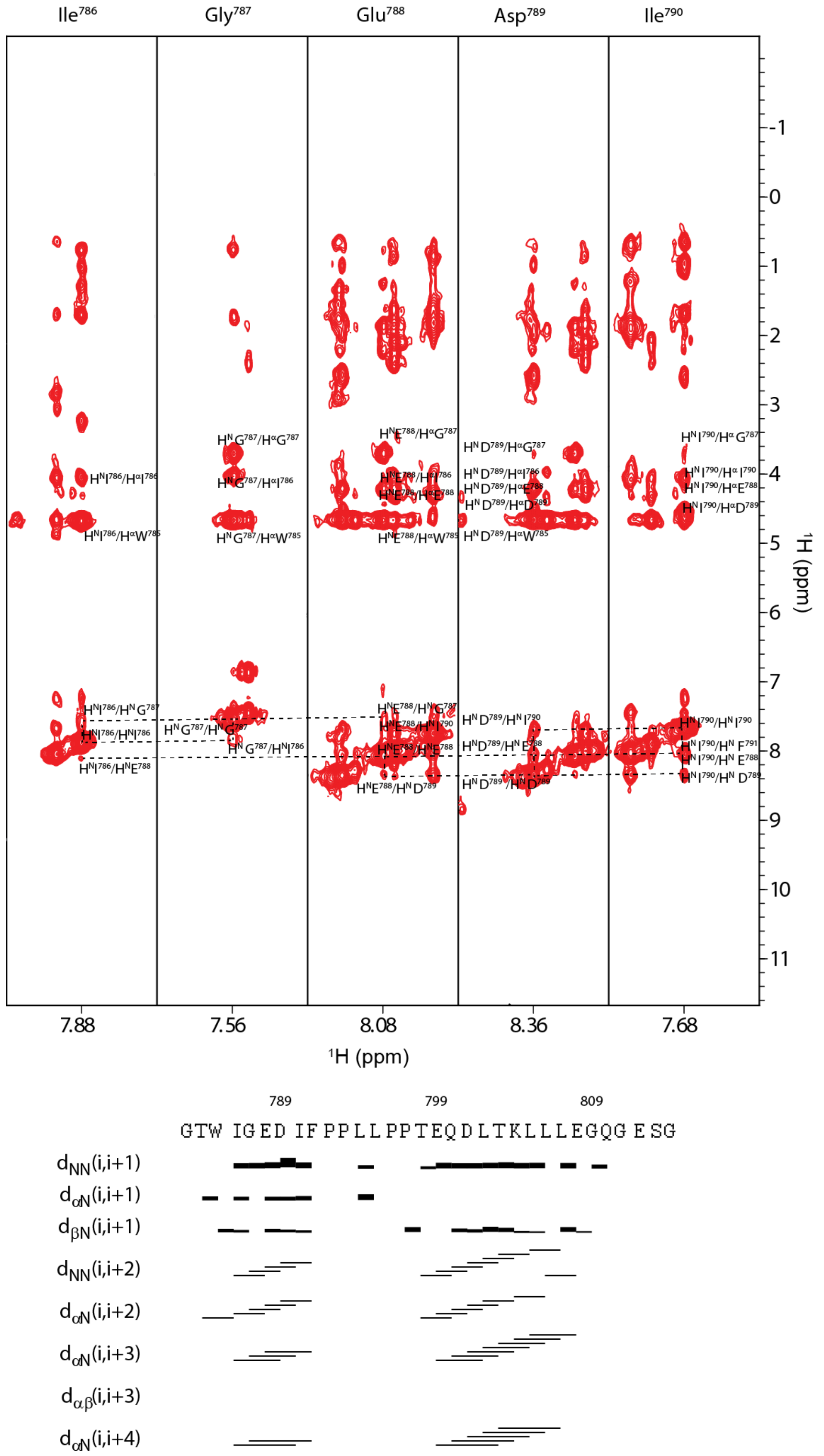

Figure SI 9. NOEs evaluation of STAT6$^{783\text{-}814}$ in complex with the PAS-B domain.

(Upper) Strips from the 3D ^{1}H,^{15}N NOESY-HSQC experiment measured on the ^{15}N-^{13}C-labeled STAT6$^{783\text{-}814}$ in complex with the PAS-B domain. The strips are related to the residues Ile^{786}, Gly^{787}, Glu^{788}, Asp^{789} and Ile^{790} located in the N terminal region of the peptide and the sequential NOEs are reported. *(Lower)* Summary of the main short and medium range NOEs observed for the STAT6$^{783\text{-}814}$ in complex with the PAS-B domain.

References

1 Brunger, A. T. Free R value: a novel statistical quantity for assessing the accuracy of crystal structures. *Nature* **355**, 472-475 (1992).

2 Broennimann, C. *et al.* The PILATUS 1M detector. *J Synchrotron Radiat* **13**, 120-130 (2006).

3 Kabsch, W. Xds. *Acta Crystallogr D Biol Crystallogr* **66**, 125-132 (2010).

4 McCoy, A. J. Solving structures of protein complexes by molecular replacement with Phaser. *Acta Crystallogr D Biol Crystallogr* **63**, 32-41 (2007).

5 Razeto, A. *et al.* Structure of the NCoA-1/SRC-1 PAS-B domain bound to the LXXLL motif of the STAT6 transactivation domain. *J Mol Biol* **336**, 319-329 (2004).

6 Murshudov, G. N. *et al.* REFMAC5 for the refinement of macromolecular crystal structures. *Acta Crystallogr D Biol Crystallogr* **67**, 355-367 (2011).

7 Emsley, P., Lohkamp, B., Scott, W. G. & Cowtan, K. Features and development of Coot. *Acta Crystallogr D Biol Crystallogr* **66**, 486-501 (2010).
